# Plant biomechanics and resilience to environmental changes are controlled by specific lignin chemistries in each vascular cell type and morphotype

**DOI:** 10.1093/plcell/koac284

**Published:** 2022-09-21

**Authors:** Delphine Ménard, Leonard Blaschek, Konstantin Kriechbaum, Cheng Choo Lee, Henrik Serk, Chuantao Zhu, Alexander Lyubartsev, Nuoendagula, Zoltán Bacsik, Lennart Bergström, Aji Mathew, Shinya Kajita, Edouard Pesquet

**Affiliations:** Department of Ecology, Environment and Plant Sciences (DEEP), Stockholm University, 106 91 Stockholm, Sweden; Umeå Plant Science Centre (UPSC), Department of Plant Physiology, Umeå University, 901 87 Umeå, Sweden; Department of Ecology, Environment and Plant Sciences (DEEP), Stockholm University, 106 91 Stockholm, Sweden; Department of Materials and Environmental Chemistry (MMK), Stockholm University, 106 91 Stockholm, Sweden; Umeå Core Facility for Electron Microscopy (UCEM), Umeå University, 901 87 Umeå, Sweden; Umeå Plant Science Centre (UPSC), Department of Plant Physiology, Umeå University, 901 87 Umeå, Sweden; Department of Materials and Environmental Chemistry (MMK), Stockholm University, 106 91 Stockholm, Sweden; Department of Materials and Environmental Chemistry (MMK), Stockholm University, 106 91 Stockholm, Sweden; Graduate School of Bio-Applications and Systems Engineering, Tokyo University of Agriculture and Technology, Tokyo 184-8588, Japan; Department of Materials and Environmental Chemistry (MMK), Stockholm University, 106 91 Stockholm, Sweden; Department of Materials and Environmental Chemistry (MMK), Stockholm University, 106 91 Stockholm, Sweden; Department of Materials and Environmental Chemistry (MMK), Stockholm University, 106 91 Stockholm, Sweden; Graduate School of Bio-Applications and Systems Engineering, Tokyo University of Agriculture and Technology, Tokyo 184-8588, Japan; Department of Ecology, Environment and Plant Sciences (DEEP), Stockholm University, 106 91 Stockholm, Sweden; Umeå Plant Science Centre (UPSC), Department of Plant Physiology, Umeå University, 901 87 Umeå, Sweden; Bolin Centre for Climate Research, Stockholm University, 106 91 Stockholm, Sweden

## Abstract

The biopolymer lignin is deposited in the cell walls of vascular cells and is essential for long-distance water conduction and structural support in plants. Different vascular cell types contain distinct and conserved lignin chemistries, each with specific aromatic and aliphatic substitutions. Yet, the biological role of this conserved and specific lignin chemistry in each cell type remains unclear. Here, we investigated the roles of this lignin biochemical specificity for cellular functions by producing single cell analyses for three cell morphotypes of tracheary elements, which all allow sap conduction but differ in their morphology. We determined that specific lignin chemistries accumulate in each cell type. Moreover, lignin accumulated dynamically, increasing in quantity and changing in composition, to alter the cell wall biomechanics during cell maturation. For similar aromatic substitutions, residues with alcohol aliphatic functions increased stiffness whereas aldehydes increased flexibility of the cell wall. Modifying this lignin biochemical specificity and the sequence of its formation impaired the cell wall biomechanics of each morphotype and consequently hindered sap conduction and drought recovery. Together, our results demonstrate that each sap-conducting vascular cell type distinctly controls their lignin biochemistry to adjust their biomechanics and hydraulic properties to face developmental and environmental constraints.

## Teaser

During the development of each vascular cell, specific lignin chemistries control their biomechanics and water conduction properties to face environmental changes.

IN A NUTSHELL **Background:** Lignin comprises multiple cell wall–localized aromatic polymers that are essential for vascular plants to conduct water and strengthen their organs. It has long been thought that lignin was randomly and nonspecifically assembled to provide mechanical strengthening and waterproofing to cells by filling-up the empty spaces in the cell walls. However, the different cell types and morphotypes forming the different sap-conducting pipes and their cell wall layers (inner vs. outer layer) exhibit specific lignin chemistries that are conserved among plant species. We, therefore, investigated the function of these specific lignin chemistries at the cell and cell wall layer levels for the different sap-conducting pipes in plants.

**Question:** What is the function of a specific lignin chemistry for the different plant sap-conducting pipe cells? Can changes in the lignin chemistry of sap-conducting cells affect their hydraulic capacity when facing environmental conditions such as drought?

**Findings:** We answered these questions by changing lignin levels and composition, using drugs to block lignin formation, and/or genetic engineering to switch off genes, in three complementary systems: (1) isolated cells grown in test tubes that we can trigger to become sap conduits, (2) annual plants, and (3) hardwood trees. We show that lignin chemistry is specific to each cell morphotype and changes during cell maturation, modifying the amount of lignin, the chemical composition of lignin units, and the position of these units in the longer polymer. These specific lignin chemistries are required for the proper function of each cell morphotype to properly conduct the sap and strengthen plant organs. Modifying the amount, the composition, and the time when specific units with distinct chemistry are incorporated in lignin of each cell morphotype has dramatic effects, causing defects in sap conduit hydraulic and biomechanical properties. The ratio between the different chemical units of lignin needs to be fine-tuned to adjust plant sap conduction and mechanical strengthening. Thus, changes in the proportion of lignin units with distinct chemistries confer different hydraulic and mechanical properties enabling plants to better resist and/or recover from drought. We also revealed that increases in the proportion of lignin units with aldehyde modulate plant pipe hydraulic and mechanical properties.

**Next steps:** We are now working to identify and understand the molecular mechanisms that control the formation of specific lignin chemistries in distinct sites and times during the development of the different cell wall layers in each cell type and morphotype.

## Introduction

Vascular plants have a unique tissue called xylem that functions as both a load-bearing skeleton and a conductive system for long-distance water transport. This dual function depends on the xylem conduit cells called tracheary elements (TEs). During their differentiation, TEs reinforce their primary cell walls (PCWs) with patterned secondary cell walls (SCWs), and subsequently remove their intracellular contents by programmed cell death. This hollowing out of TEs triggers their water conducting function, forming unobstructed tubes with thickened and regularly patterned sides (Derbyshire et al., 2015; Ménard et al., 2015). As the plant grows, new TEs form, die, and connect both longitudinally and laterally to other TEs to conduct water throughout the plant (Ménard and Pesquet, 2015).

Because TEs require cell death to function, they have been considered inert and unable to adjust to changing developmental and environmental constraints. However, TE cell walls undergo modifications after their death, such as a continuous accumulation of lignin (Pesquet et al., 2010, 2013; Smith et al., 2013; Derbyshire et al., 2015; Blaschek et al., 2020a). This postmortem lignification is catalyzed by oxidative enzymes embedded in the TE cell walls and a cooperative supply of lignin monomers by surrounding cells (Barros et al., 2015; Blaschek et al., 2022). The oxidative polymerization of lignin fills the gaps between the cellulose and hemicellulose polymers in the cell walls formed before TE cell death (Blaschek and Pesquet, 2021).

To respond to developmental and environmental changes, the xylem forms different TE morphotypes with specific dimensions and SCW patterns such as narrow protoxylem (PX) with annular or spiral patterns, wide metaxylem (MX) with reticulate or pitted patterns, or later forming secondary xylem (SX) with SCW patterning similar to the MX but with a narrower diameter. Additionally, the xylem also changes the cell types surrounding each TE morphotype, varying the proportion of neighboring TEs, unlignified xylem parenchyma (XP), and lignified xylary fibers (XFs; Supplemental Figure S1; Chaffey et al., 2002; Derbyshire et al., 2015). The xylem sap, mainly consisting of water, ascends in the lumens of the dead interconnected TEs due to a negative pressure pull caused by gradients of water potential (Ψ) along the soil–plant–atmosphere continuum, and is finally released by evapotranspiration through the leaves. Unlike the Ψ of soil and air that vary with environmental conditions (Supplemental Figure S1), plants actively control their Ψ using both stomatal movements to regulate leaf transpiration rates, and the intracellular osmolarity of XPs and XFs to alter osmotic pressure (Holbrook et al., 1995; Pockman et al., 1995; Bentrup, 2017). The optimal TE morphology for laminar sap flow is a cylindrical pipe, according to the Hagen–Poiseuille law (Calkin et al., 1986; Tyree et al., 1994; Venturas et al., 2017). However, TEs cannot withstand very large Ψ differences such as those encountered during drought, which cause TEs to collapse inwardly, altering their circularity and consequently disrupting plant hydraulic conductivity (Supplemental Figure S1; Cochard et al., 2004; Brodribb and Holbrook, 2005; Coleman et al., 2008; Kitin et al., 2010; Voelker et al., 2011; Zhang et al., 2016). TE inward collapse is sometimes reversible, acting as a circuit breaker to resist extreme drought, which is restored once water availability improves (Zhang et al., 2016).

TE inward collapse is also observed when modifying the biosynthesis of TE SCWs using genetic mutations (Turner and Sommerville, 1997; Brown et al., 2005) or drug treatment (Amrhein et al., 1983; Smart and Amrhein, 1985) where it is called irregular xylem (*irx*). This *irx* phenotype is only observed in dead sap-conducting TEs but never in non-sap-conducting TEs produced either in isolated suspension cells (Endo et al., 2008) or ectopically in nonxylem tissues (Takenaka et al., 2018). The *irx* phenotype reveals the importance of TE cell wall composition, concentration, and/or structure to establish a mechanical resistance sufficient to cope with Ψ variations from the soil and the atmosphere.

Lignin formation is genetically controlled and affects the concentration, composition, and structure of lignin polymers during the development and stress response for each cell type in their different cell wall layers (Blaschek et al., 2020a, 2020b; Yamamoto et al., 2020; Hiraide et al., 2021). The main lignin residues are C_6_C_3_ phenylpropanoids (Supplemental Table S1; Moss, 2000) that differ by their C_6_ aromatic *meta* groups, such as monomethoxylated guaiacyl (G) and dimethoxylated syringyl (S) rings, and in their C_3_ aliphatic terminal functions, such as alcohol (X_CHOH_) or aldehyde (X_CHO_; Dixon and Barros, 2019). TEs accumulate mostly G residues in their cell walls independently of the plant species (Pesquet et al., 2019). Distinct TE morphotypes also differently accumulate X_CHO_ residues (Yamamoto et al., 2020). Additionally, noncanonical residues are also differently incorporated in lignin depending on tissues and plant species, such as benzaldehydes (Ralph et al., 2001; Kim et al., 2003), coumaroyl esters (Lapierre et al., 2021), stilbenoids (del Río et al., 2017; Rencoret et al., 2019), flavonoids (Lan et al., 2015; Rencoret et al., 2022), and other residues presenting phenyl (P) rings (Faix and Meier, 1989; Kawamoto, 2017). Yet, the role of this conserved cell and morphotype-specific lignin chemistry for TEs is still not understood.

Here, we investigated the biological roles of the specific lignin chemistry of the different TE morphotypes on plant load-bearing and vascular properties. We used three complementary biological systems to fully investigate lignin in TEs: (1) an inducible plant pluripotent cell suspension culture (iPSC) from Arabidopsis (*Arabidopsis thaliana*), (2) annual herbaceous Arabidopsis plants with genetically altered lignins, and (3) perennial woody poplar (*Populus tremula×tremuloides*) plants with genetically altered lignins. IPSCs enable the modification of lignin in isolated TE morphotypes at distinct postmortem maturation stages and the investigation of the role played by lignin in isolated TEs independently of the physiological constraints of sap conduction or tissue pressure. Genetically modified plants with altered lignin amounts and compositions facilitate the assessment of the role of specific lignin chemistry on the different sap-conducting TE morphotypes embedded in functional vascular tissues. Perennial poplar plants additionally enable the monitoring of the dendrochronological changes of lignin in TEs within woody tissues during secondary growth. Using these complementary systems, we demonstrated that different TE morphotypes accumulate specific lignins during postmortem lignification for optimal hydraulic properties. More specifically, the proportions of different C_3_ terminal functions for the same C_6_ substitution balanced cell wall stiffness with flexibility. Together, our study suggests that lignin structure is specifically fine-tuned during the postmortem maturation of each functioning TE morphotypes to dynamically adjust their conductive and load-bearing properties to changing developmental and environmental conditions.

## Results

### Postmortem accumulation of lignin increases the cell wall stiffness of isolated TEs morphotypes

**Figure 1:**
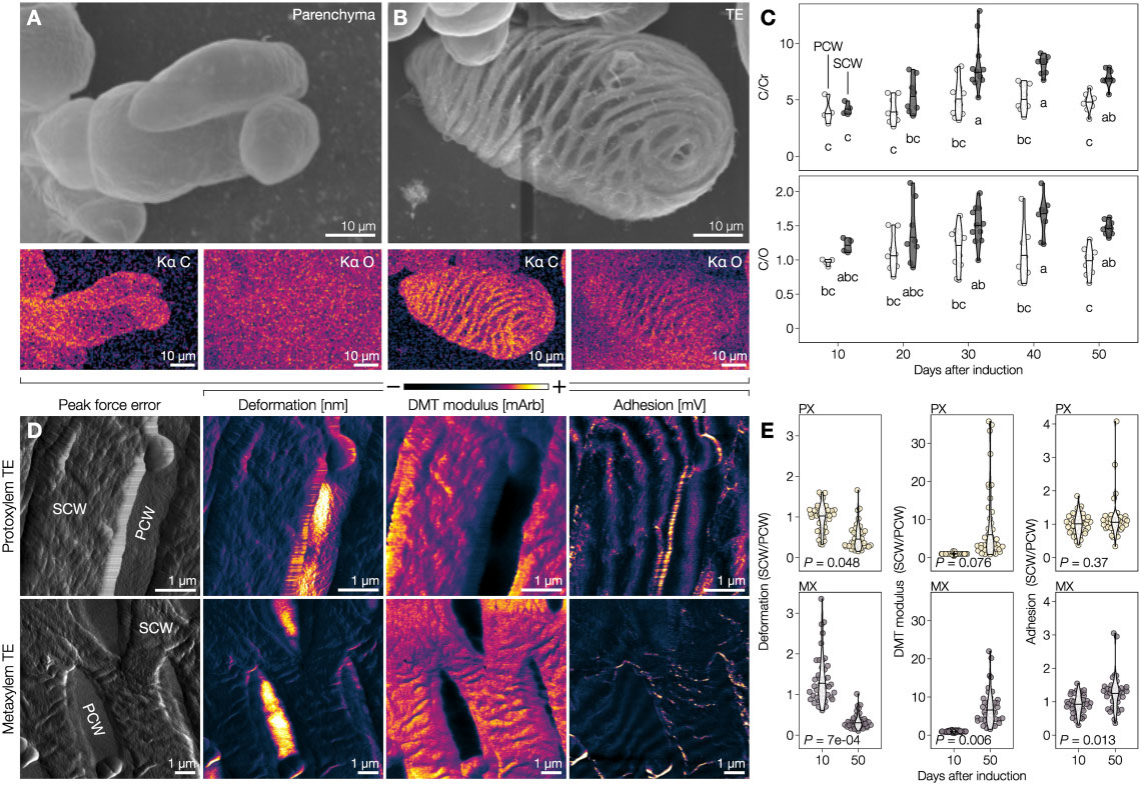
Postmortem lignification actively alters biomechanics of TE SCWs. A, Scanning electron micrograph of isolated parenchyma cells, prepared using critical-point drying (CPD), as well as their EDS carbon (C) and oxygen (O) signals in color-coded intensity. B, Scanning electron micrograph of an isolated TE 30 d after induction, prepared by CPD, with color-coded intensity EDS C and O signals. C, EDS ratios of C to coating chromium (Cr) and C to O ratios of 9 µm^2^ (300 nm × 300 nm) of the primary (PCW) and SCW from isolated 10- to 50-d-old TEs. Note that SCWs gradually increase their C/O ratio during postmortem maturation; *n *=* *4–11 individual cells per time point. Different lowercase letters indicate significant differences according to a Tukey-HSD test (per panel; α = 0.05). D, AFM peak force error and intensity color-coding of deformation, DMT modulus, and adhesion of 50-d-old isolated PX and MX TEs. We used mArb and mV as arbitrary units to report changes for stiffness and adhesion, respectively, to the cantilever between independent cells measured at different times. E, SCW to PCW ratios of deformation, DMT modulus, and adhesion of 10- and 50-d-old PX and MX TEs. The *P*-value of a two-tailed Welch’s *t* test is indicated; *n *=* *4 individual cells per time point and morphotype, 10 measurements per cell.

To define the influence of lignin accumulation on the cell walls of isolated TEs, we used iPSCs as they make it possible to follow the maturation of intact single-cell TEs that cannot easily be isolated from whole plants using maceration or dissection (Ménard et al., 2017). IPSCs from Arabidopsis synchronously produced all TE morphotypes, which underwent cell death 5–7 d after induction, followed by postmortem lignification (Pesquet et al., 2010, 2013; Derbyshire et al., 2015). We monitored these postmortem changes in TEs at the nanoscale level using scanning electron microscopy (SEM) coupled with energy-dispersive X-ray spectroscopy (EDS) to measure elemental changes in cell wall composition (Figure 1, A and B). Ratios of carbon (C) to coating chromium (Cr) contents showed gradual postmortem increases of C-rich compounds only in SCWs compared to PCWs, which plateaued by Days 40–50 (Figure 1C). Ratios of carbon to oxygen (O) contents revealed that the compounds accumulating postmortem in TE SCWs are lowly oxygenated as expected from lignin (9 C:3–5 O for lignin-units compared to 6 C:6 O for cellulose units; Figure 1C). Once the plateau was reached, PX TEs presented SCWs with significantly less C/Cr and C/O contents than MX TEs, indicating differences in cell wall polymer amounts and composition between TE morphotypes (Supplemental Figure S2). Complementary biochemical analyses confirmed that the postmortem changes in cell wall correspond to increases in lignin concentrations rising gradually only in TE cells, reaching up to 25% of total dry cell wall weight by Day 30 (Supplemental Figure S2). We then evaluated the effect of postmortem lignification on TE biomechanics using atomic force microscopy (AFM) on 5–20 µm^2^ areas of isolated 10- and 50-d-old PX and MX TEs (Figure 1D). AFM analysis showed that postmortem lignification has almost no impact on PCWs but did alter the biomechanics of SCWs for each TE morphotype by significantly decreasing deformability for both TE morphotypes as well as increasing stiffness and adhesion for MX (Figure 1E). We conclude that SCWs of each TE morphotype continue lignifying for more than 40 d after cell death to accumulate specific amounts of lignin to increase the stiffness of TE SCWs.

### Postmortem lignin accumulation controls the resistance of isolated TE morphotypes to negative pressure

**Figure 2:**
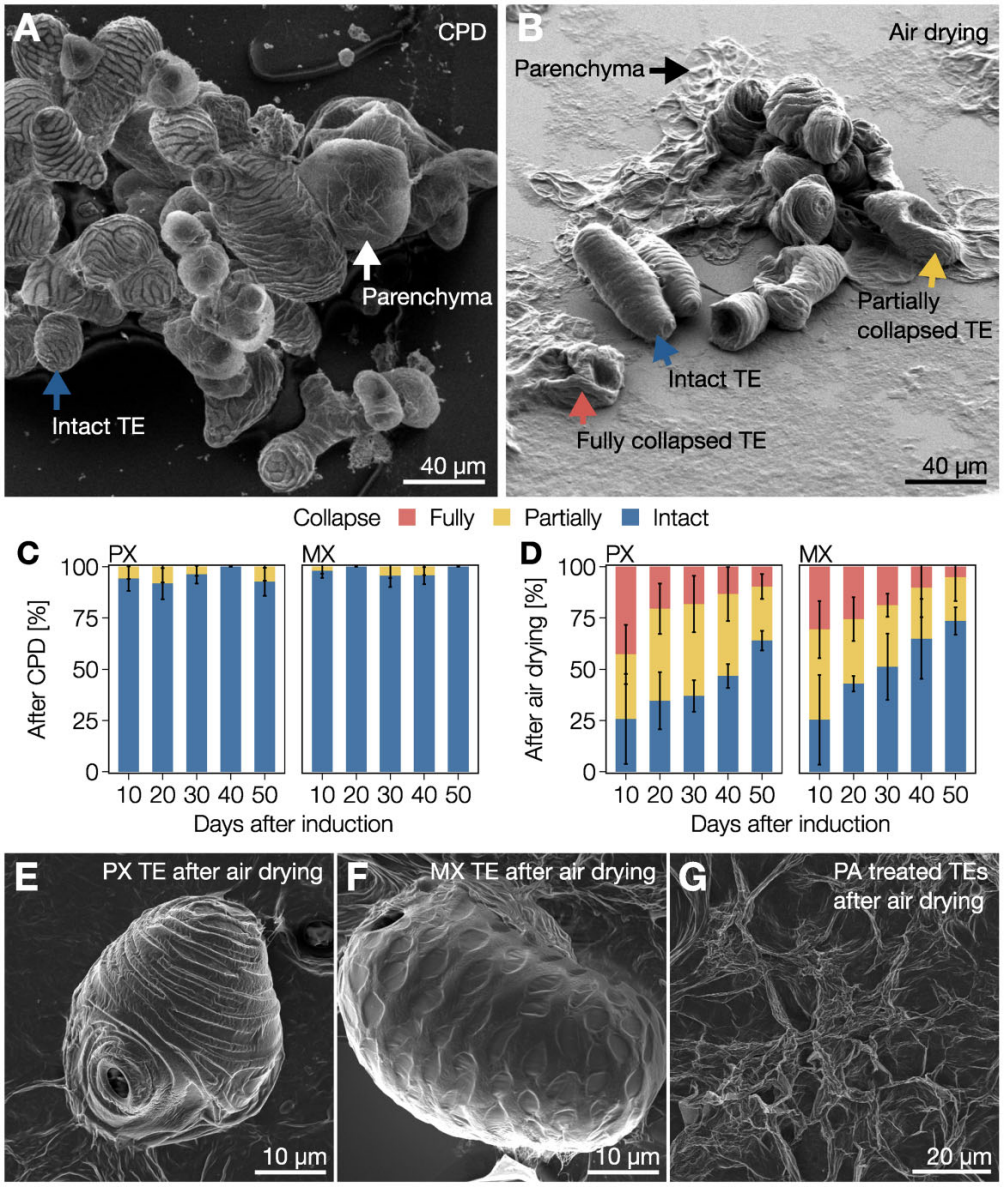
Gradual postmortem lignification enables all TE morphotypes to resist extreme Ψ differentials. A, Scanning electron micrograph of 30-d-old isolated TEs and parenchyma cells produced from iPSCs and prepared using CPD. Note that both TEs and parenchyma are intact, as indicated by the blue and white arrows, respectively. B, Scanning electron micrograph of 30-d-old isolated TEs and parenchyma cells produced from iPSCs and prepared using air drying. Note that parenchyma cells (black arrow) are completely flattened whereas TEs were either fully collapsed (red arrow), partially collapsed (yellow arrow), or intact (blue arrow). C and D, Relative proportion of 10- to 50-d-old TEs from iPSCs that were fully collapsed, partially collapsed, or intact after CPD (C) or air drying (D). Error bars represent ± SD of three independent experiments; *n *=* *27–159 individual cells per cell type and time point. E, Scanning electron micrograph of a 30-d-old PX TE after air drying. F, Scanning electron micrograph of a 30-d-old MX TE after air drying. G, Scanning electron micrograph of 30-d-old unlignified TEs treated with PA after air drying.

Next, we evaluated the role of stiffness increases due to postmortem lignification in TE resistance to negative pressures such as those faced during water conduction. To this end, we exposed isolated 10- and 50-d-old TEs to two different drying methods followed by observation using SEM. We compared critical-point drying (CPD), which minimizes negative pressure differences during drying, to air drying, which mimics drought by exposing the cells to large Ψ differences. Parenchymatic cells showed no inward collapse after CPD but were completely flattened by air drying (Figure 2, A and B). TEs were similarly unaffected by CPD but partly withstood air drying (Figure 2, A and B). These results showed that TE collapse occurs in response to the negative pressure exerted on single TEs itself and is not due to the surrounding tissue pressure. Analysis of the proportion of collapsed TEs after air drying during postmortem lignification revealed a gradual increase of resistance to collapse, with the majority of 50-d-old TEs remaining completely intact (Figure 2, C and D). As the susceptibility to collapse lowered as postmortem lignification increased, our results moreover indicated that TE susceptibility to collapse did not depend on the SCW polysaccharidic polymers deposited pre-mortem but rather on the free spaces in cell walls defined for lignification. Both PX and MX TEs (Figure 2, E and F) withstood collapse in air drying better as postmortem lignification progressed, although PX were consistently more sensitive than MX TEs (Figure 2, C and D). To ascertain that lignins caused the observed increased resistance, we obtained TEs with SCW devoid of any lignin by treating iPSCs with piperonylic acid (PA), an inhibitor of the CINNAMATE-4-HYDROXYLASE (C4H) enzyme controlling C_6_C_3_ biosynthesis (Supplemental Figure S2; Pesquet et al., 2013; Van de Wouwer et al., 2016; Decou et al., 2017). Analysis of lignin content following PA-treatment confirmed that treated TEs did not accumulate any lignin, neither pre- nor postmortem (Supplemental Figure S2). The resulting unlignified TEs completely collapsed with air drying (Figure 2G). These results demonstrate that lignin amounts in SCWs control TE biomechanics in isolated cells to resist collapse. Therefore, the role of postmortem lignification in TEs is to dynamically reinforce the cell walls of TEs to sustain changes in negative pressure gradients.

### Each TE morphotype differs in morphology, lignin concentration, and composition in annual plants

**Figure 3:**
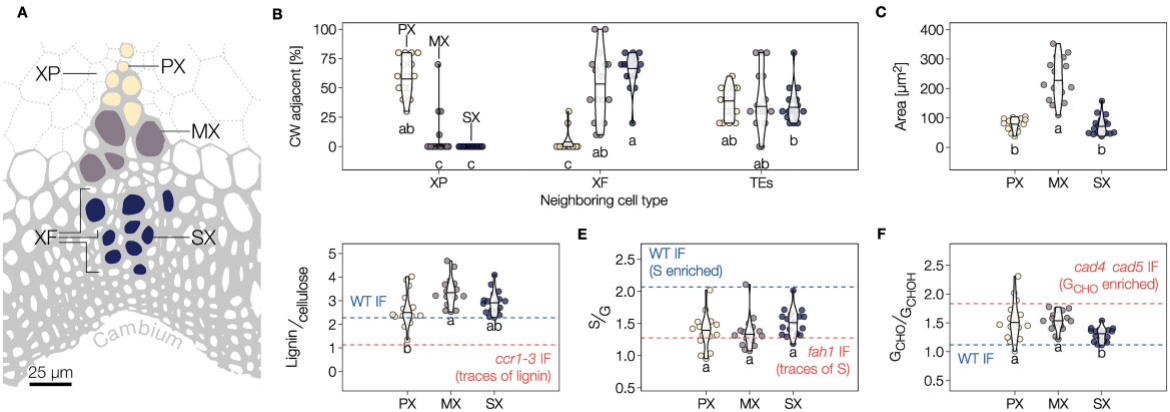
Different TE morphotypes in annual plants have specific morphological features and lignin chemistry. A, Schematic diagram of the localization of the three TE types in vascular bundles of Arabidopsis stems according to their distance to the cambium: PX in yellow, MX in purple and SX in blue. Each TE morphotype shares different proportions of XFs and XP surrounding each TE type. B, Relative proportion of adjacent cell types for each TE morphotype. Note that the proportion of neighboring TEs remains constant independently of the TE morphotype. Different lowercase letters indicate significant differences according to a Kruskal–Wallis test followed by Dunn’s multiple comparison (α = 0.05). C, Lumen area of each TE morphotype determined from cross-sections. D, Relative lignin to cellulose ratio measured by Raman microspectroscopy. E, Relative S to G ratio measured by Raman microspectroscopy. F, Relative G_CHO_ to G_CHOH_ ratio measured by Raman microspectroscopy. Because range and intercept of Raman band ratios differ from other biochemical analyses but still maintain a linear relationship (Agarwal, 2019; Blaschek et al., 2020b), references are presented for each lignin parameter using interfascicular fibers (IF) in WT and relevant mutants. Different lowercase letters in panels C–H indicate significant differences according to a Tukey-HSD test (per panel; α = 0.05); *n *=* *15–17 individual cells per TE type in three plants.

We then investigated the role of lignification on the collapse of TEs embedded in tissues using the herbaceous plant Arabidopsis. Three TE morphotypes are present in fully grown 8-week-old stems of wild-type (WT) plants prior to senescence, identifiable by their distance to the cambium: PX TEs, MX TEs, and secondary xylem TEs (SX; Figure 3A). For all morphotypes, cell types directly adjacent to each TE included always ∼35% of other TEs but various proportions of XP and XF (Figure 3B). PX and SX had smaller lumen diameters compared to MX (Figure 3C). We performed a semi-quantitative lignin analysis of each TE morphotype using Raman spectroscopy and calibrated it to key lignin mutants (Supplemental Figure S3; Blaschek et al., 2020b). MXs presented higher lignin levels than PX (Figure 3D). All TEs exhibited similar proportions of S/G residue (Figure 3E). The ratio of terminal coniferaldehyde (G_CHO_) to total coniferyl alcohol (G_CHOH_; including terminal coniferyl alcohol as well as internal guaiacylglycerol, pinoresinol, and other incorporated G_CHOH_ structures) was lower in SX than in PX or MX (Figure 3F). The proportion of noncanonical benzaldehyde and P residues also varied between morphotypes, increasing in PX compared to MX and SX (Supplemental Figure S4). Total G_CHO_, as measured using the Wiesner test (Blaschek et al., 2020a), showed that PX accumulates less G_CHO_ than MX and SX (Supplemental Figure S4). Overall, each TE morphotype had specific dimensions, SCW organization, and neighboring cells as well as distinct lignin composition, amounts, and structure.

### Lignin concentration and composition differently affects the resistance to negative pressure of each TE morphotype in annual plants

**Figure 4:**
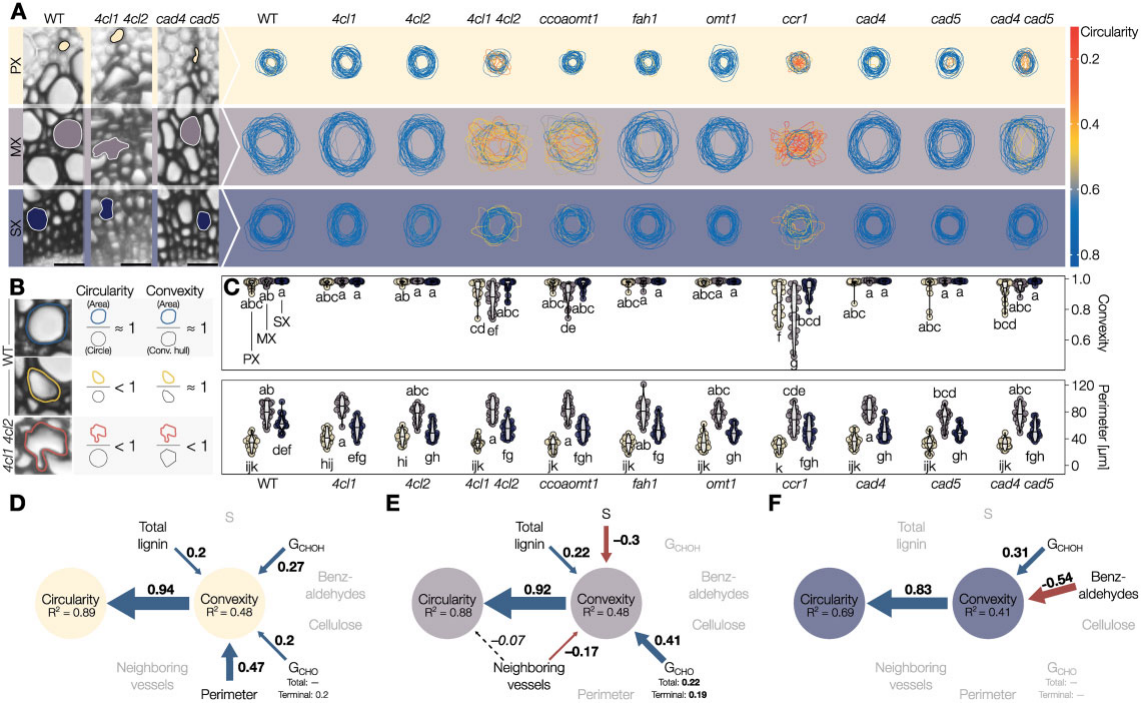
Lignin structure differently alters the resistance of specific TE morphotypes in annual plants. A, Traces of 25 representative perimeters for each TE type in transverse cross-sections from stems of Arabidopsis loss-of-function mutants altered in lignin structure. The outline color indicates the circularity of each respective TE. B, Schematic explanation of circularity and convexity of TEs. Any deviation from a perfect circle will decrease circularity, whereas only inward collapse of the perimeter will decrease convexity. C, Convexity and perimeter of PX, MX, and SX TEs in different phenylpropanoid biosynthesis mutants; *n *=* *50 TEs per morphotype and genotype. Different lowercase letters indicate significant differences according to a Tukey-HSD test (per panel; α = 0.05). D–F, Structural equation models of the factors influencing TE convexity and circularity in the PX (D), MX (E), and SX (F) of Arabidopsis. Blue arrows and positive standardized coefficients indicate significant positive effects, red arrows and negative standardized coefficients indicate significant negative effects. Dashed arrows indicate predictors that were included and improved the model, but whose specific effects were not statistically significant. Grayed out variables had no significant effect on TE convexity.

To determine the importance of accumulating specific lignins for the mechanical resistance of sap-transporting TEs, we evaluated TE lignification and collapse in nine loss-of-function Arabidopsis mutants altered in their lignin concentration and/or composition (list of genes mutated is provided in Supplemental Table S2). We measured TE morphology and lignin structure using in situ quantitative chemical imaging (Blaschek et al., 2020a, 2020b; Yamamoto et al., 2020) of fully grown 8-week-old stems. For TE morphology, we measured inward collapse, estimated by a decreased convexity, and deformation, shown by a reduction of circularity (general deviation from a perfect circular shape) compared to TEs in WT plants (Figure 4, A and B). The mutant array employed here provided a dataset of around 100 TEs per morphotype with a wide and continuous variation in convexity and lignin structure (Supplemental Figure S5), suitable for the detection of specific associations between lignin amount/composition and TE resistance to collapse for each morphotype. TE perimeter and neighboring cell types remained unaltered in all TE morphotypes between mutants, thereby confirming that neither TE pre-mortem formation nor surrounding cells were altered by these mutations (Supplemental Figure S4). By contrast, the convexity and circularity of specific TE morphotypes were reduced by genetic changes leading to various degrees of deformation (Figure 4C): unaffected in most genotypes, all TE morphotypes were altered in *ccr1* (defective in CINNAMOYL COA REDUCTASE 1) and *4cl1 4cl2* (lacking activity for two 4-COUMARATE-COA LIGASE enzymes) whereas *ccoaomt1* (defective in CAFFEOYL COA O-METHYLTRANSFERASE 1) only altered MXs and *cad4 cad5* (lacking CINNAMYL ALCOHOL DEHYDROGENASE activity from CAD4 and CAD5) only PXs (Figure 4C). As each mutation causes many biochemical changes in lignin, some overlapping and others distinct (Supplemental Figure S4), we used structural equation models to identify significant associations between the lignin chemical parameters and the inward collapse of each TE morphotype. These models showed that changes in lignin, but not cellulose, are associated with changes in resistance of TE collapse independently of the morphotype (Figure 4D), confirming the role of postmortem lignin accumulation in TEs to resist negative pressure, as observed in isolated TEs using iPSCs (Figure 2). In addition to the positive effect of higher lignin amounts, models showed that increases in terminal G_CHO_, total G_CHOH_ contents, and TE perimeter are associated with higher resistance to collapse in PXs (Figure 4D). MX resistance to collapse was similarly associated with higher levels of total lignin and G_CHO_ (both total and terminal), whereas increases in S residues and in the proportion of neighboring TEs decreased their resistance to collapse (Figure 4E). The association between increases in lignin and lower susceptibility to TE collapse for both PX and MX directly confirmed our observations of isolated TEs from iPSCs (Figure 2). TE collapse was rare in SXs (Figure 4A) and only associated with lower levels of G_CHOH_ and high levels of benzaldehydes (Figure 4F). The models for each TE morphotype were further improved by investigating the interaction effects between morphological and biochemical features (Supplemental Figure S5). In PXs, we observed that the synergistic strengthening effects of increased levels of G_CHOH_ and G_CHO_ promoting TE resistance to collapse depend on TE perimeter, with a narrow PX benefiting more from these residue increases than wider PXs (Supplemental Figure S5). In MXs, the positive effect of higher G_CHO_ was synergistic with total lignin amounts to promote TE resistance, but this effect depended on neighboring TE proportions, with isolated MXs benefiting more from these residue increases than grouped MXs (Supplemental Figure S5). Overall, TE susceptibility to collapse in each TE morphotype was associated with specific changes and interactions between cell/tissue morphology and lignin chemistry (amount, S/G and G_CHO_/G_CHOH_ compositional ratios, G_CHO_ terminal to total). Our results established that the different lignin residues have nonredundant roles and need to be specifically controlled for each TE morphotype to sustain negative pressure for optimal water conduction.

### Postmortem incorporation of specific lignin residues alters TE resistance during wood formation in perennial plants

**Figure 5:**
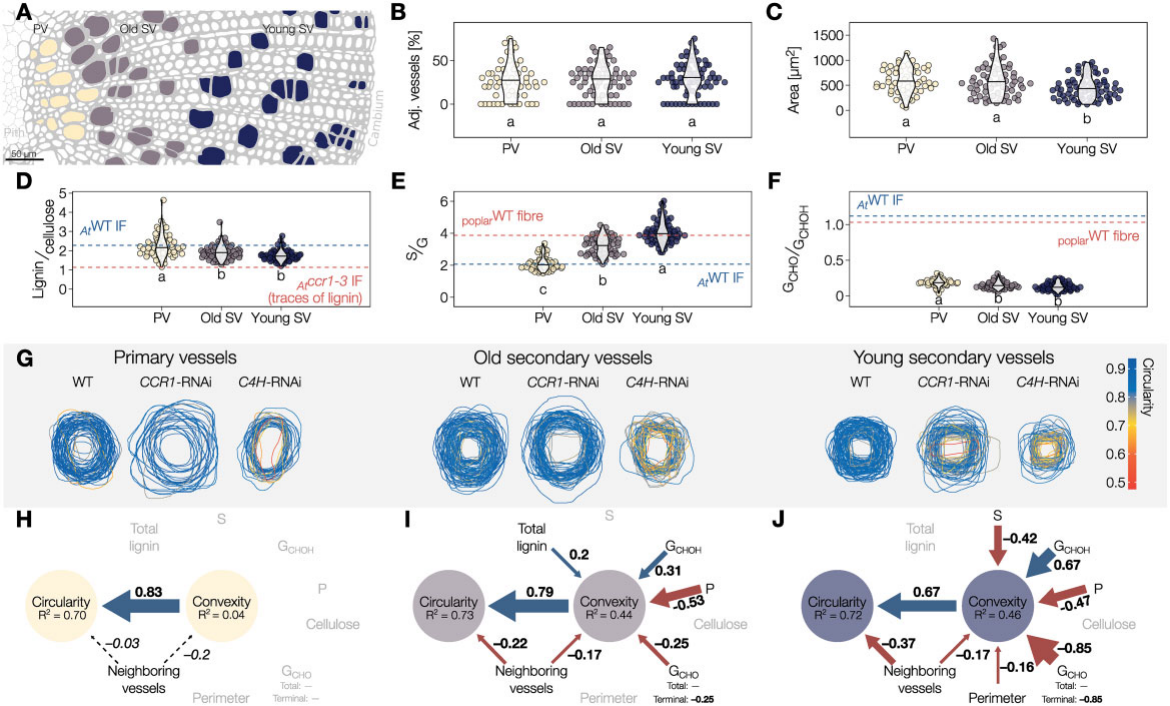
Different TE morphotypes in woody plants depend on specific postmortem accumulated lignins for their resistance against collapse. A, Schematic diagram of the three TE types in the xylem of poplar stems, oriented on the pith–cambium axis: primary vessels (PV) in yellow, old secondary vessels (old SV) in purple, and young secondary vessels (young SV) in blue. B, Relative proportion of adjacent TEs for each TE type. Note that the proportion of TEs neighboring other TEs is independent of TE type and very similar to the proportions in Arabidopsis. C, Lumen area of each TE type determined from cross-sections. D, Relative lignin to cellulose ratio measured by Raman microspectroscopy. E, Relative S to G ratio measured by Raman microspectroscopy. F, Relative G_CHO_ to G_CHOH_ ratio measured by Raman microspectroscopy. Because range and intercept of Raman band ratios differ from other biochemical analyses but still maintain a linear relationship (Agarwal, 2019; Blaschek et al., 2020b), references are presented for each lignin parameter using poplar fibers or the interfascicular fibers (IF) of Arabidopsis WT and relevant mutants. Different lowercase letters in panels B–F indicate significant differences according to a Tukey-HSD test (per panel; α = 0.05); *n *=* *56–72 individual cells from five plants per TE type. G, Representation of TE perimeter for each TE type in transverse cross-sections from stems of *Populus tremula×tremuloides* RNAi plants altering lignin biosynthesis. TE outline color indicates the circularity of each respective TE. H–J, Structural equation models of the factors influencing TE convexity and circularity in the PV (H), old SV (I), and young SV (J). Blue arrows and positive standardized coefficients indicate significant positive effects, red arrows and negative standardized coefficients indicate significant negative effects. Dashed arrows indicated predictors that were included and improved the model, but whose specific effects were not statistically significant. Grayed out variables had no significant effect on TE convexity.

To assess the role of lignin during gradual postmortem TE maturation in tissues, we similarly evaluated TE lignification and resistance to collapse in woody whole plants of continuously growing 7- to 12-month old hybrid poplar. We focused our analyses on different TE types in stem cross-sections: primary (PV) and secondary xylem TEs/vessels (SV) at different developmental states, young and old SVs as defined by respectively being before or after the 50% distance from the cambium to the end of the wood ring (Figure 5A). All TEs in WT plants had similar surrounding cell types but varied in the lumen area, which was the smallest in young SVs (Figure 5, B and C). In situ analysis of cell wall biochemistry detected differences in lignin levels (highest in PVs) and S/G composition (highest in young SVs; Figure 5, D and E). Overall G_CHO_ levels were low, in accordance with the literature (Yamamoto et al., 2020), but the G_CHO_/G_CHOH_ ratio was higher in PVs than SVs (Figure 5F). These biochemical changes occurred gradually during wood maturation to increase lignin levels and G_CHO_/G_CHOH_ together with decreasing S/G as cell distance from the cambium increased (Supplemental Figure S6A). Overall, TE morphology differed between poplar and Arabidopsis presenting ∼3- to 4-fold larger perimeters in poplar for all morphotypes as previously shown (Chaffey et al., 2002). In contrast to Arabidopsis, the proportion of TE neighboring other TEs remained similar between morphotypes in poplar (Figure 5B). TEs in Arabidopsis had slightly higher lignin concentration compared to poplar, lower S/G but higher G_CHO_/G_CHOH_, although all TEs independently of the plant species showed high G residue enrichment (Figure 5 and Supplemental Figure S6). These observations further highlight that lignin levels and chemistry are adjusted for each TE morphotype depending on the plant species, each exhibiting differences in cell size, tissue organization, and phenolic metabolism. Altogether, these results confirm that TE postmortem lignin accumulation observed in iPSCs also occurs in wood of whole plants, and show a dynamic change of lignin chemistry during these postmortem processes as wood tissues mature. Our results moreover suggest that each plant species can modify their lignin chemistry differently to enable the function of their TE specificities.

### Lignin concentration and composition fine-tune the mechanical properties of TEs during their postmortem maturation in woody tissue

To assess the link between lignin and TE collapse in wood, we followed the same strategy as in Arabidopsis using transgenic poplar harboring RNA interference (RNAi) constructs to alter lignin amount and composition. In the three genotypes used (WT, *CCR1*-RNAi, *C4H*-RNAi), TEs also showed a wide variation in lignin structure and collapse that moreover depended on their developmental age (Figure 5G and Supplemental Figure S6). These transgenes affected the collapse of TE differently: increasing the susceptibility to collapse for all SVs in *C4H*-RNAi, altering only young SVs for *CCR1*-RNAi, whereas PVs remained unaffected (Figure 5G). Lignin levels and composition as well as the rates of lignin modification during wood maturation were also altered by the expression of these constructs, lowering the S/G gradual accumulation rate in *C4H*-RNAi whereas lowering lignin levels and G_CHO_/G_CHOH_ accumulation rates in *CCR1*-RNAi (Supplemental Figure S6). Using a single cell lignin analysis from 100 to 170 individual TEs per morphotype and development stage, we evaluated which parameters were associated with the capacity of specific TEs to withstand collapse using structural equation models. As PVs did not collapse (Figure 5H and Supplemental Figure S6C), none of the measured parameters had any effect, thereby showing unique resilience of PVs. Our structural equation models confirmed that in poplar, as in Arabidopsis, the changes in lignin and not the change in cellulose were associated with the collapse of TEs (Figure 5, I and J). In old SVs, the resistance to collapse was associated with increases of G_CHOH_ and lignin levels but was compromised by increases in terminal G_CHO_ residues and increases in neighboring TEs (Figure 5I). The resistance to collapse of young SVs was promoted by increases of G_CHOH_ but reduced by increases of S, terminal G_CHO_ residues, TE perimeter, and neighboring TEs (Figure 5J). Our results show that the relative contribution of specific lignin features depends on TE maturation stages. In contrast to the conserved negative effect of neighboring vessels on SVs, G_CHO_ residues negative contribution decreased as it accumulation increased during SV maturation (Figure 5, I and J and Supplemental Figure S6). These results suggest that lignin formation follows a specific temporal sequence of residues accumulation at distinct maturation stages to enable TEs to sustain negative pressure. Disturbing this temporal sequence, such as with an untimely accumulation of G_CHO_ residues, impaired SV capacity to resist collapse (Figure 5, I and J). Similarly to Arabidopsis, SV collapse in poplar also depended on the interaction between multiple parameters. G_CHO_ and S residues increases in young SVs synergistically increased the susceptibility to collapse independently of vessel perimeter or neighboring vessels (Supplemental Figure S7). G_CHOH_ residue increases however compensated the negative effect of high neighboring vessel amounts on old SV collapse (Supplemental Figure S7). Altogether, these results confirm that in woody tissues the different lignin residues (S/G and G_CHO_/G_CHOH_) are dynamically modulated in a specific accumulation sequence during postmortem maturation to set the biomechanics of specific TE types to resist collapse.

### Aliphatic and aromatic changes in lignin residues nonredundantly control distinct mechanical properties of plant stems

**Figure 6:**
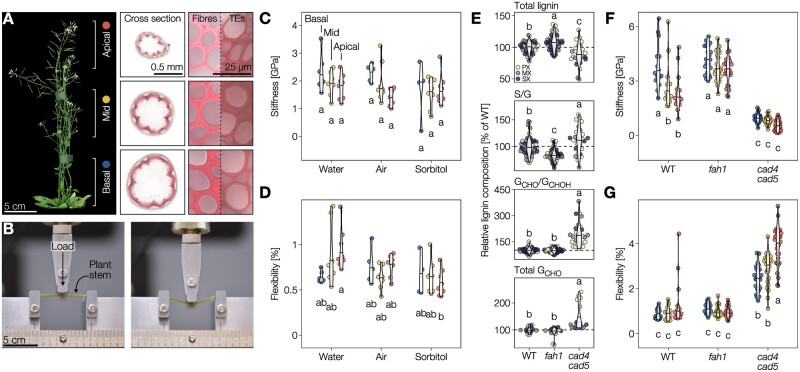
Distinct lignin monomers nonredundantly control specific mechanical properties. A, Six-week-old Arabidopsis WT plant with basal, middle, and apical stem segments showing difference in TE developmental stages and marked with the colors representing them in subsequent panels. Wiesner stained cross-sections at the bottom of each segment with close-ups of interfascicular fibers and TEs are shown. B Arabidopsis stem segment undergoing three-point bending. Flexural behavior is presented in Supplemental Movie S1. C and D, Flexural stiffness (C) and sustained elastic deformation before irreversible breaking, i.e. flexibility, (D) of WT stem segments incubated in water, air, or sorbitol determined by three-point bending; *n *=* *4–8 stem segments per developmental stage and condition. E, Total lignin, S/G, and terminal G_CHO_/total G_CHOH_ (measured by Raman microspectroscopy) and total G_CHO_ (measured using the Wiesner test) in PX, MX, and SX TEs of the different genotypes, expressed relative to the WT TEs of the respective morphotype; *n *=* *15–50 TEs per genotype. F and G, Flexural stiffness (F) and sustained elastic deformation before irreversible breaking (G) of stem segments from WT, S-depleted *fah1*, and G_CHO_-over-accumulating *cad4 cad5* mutant plants determined by three-point bending; *n *=* *14–30 stem segments per developmental stage and genotype. Different lowercase letters in panels (C–G) indicate significant differences according to a Tukey-HSD test (per panel; α = 0.05). Note that the plants for experiments shown in panels (C, D and F, G) were from different growth instances and slightly different age, explaining the slight differences in absolute stiffness.

To define how changes in lignin composition affected the biomechanics of TE cell walls and whole plants, we evaluated the effects of changing the lignin G_CHO_ to G_CHOH_ contents. The accumulation of these lignin residues showed an opposite influence on TE collapse in both herbaceous and woody plants (Figures 4 and 5) but also during TE postmortem maturation (Supplemental Figure S6). We performed three-point bending flexure measurements on Arabidopsis plant stems (Figures 6 and Supplemental Figure S8; Nakata et al., 2020). We used three different segments along the stem length, from apices to bases, to spatially separate the lignification state of TEs and other vascular cells as previously performed (Figure 6A; Pesquet et al., 2013; Hoffmann et al., 2020; Morel et al., 2022). We first evaluated the influence of turgor pressure and sap/water content on the flexure of 5- to 6-week-old stem segments after incubation in air, pure water, or 1 M sorbitol for several hours. We used Euler–Bernoulli’s equation, requiring measurements of the width of each stem segments (Supplemental Figure S8), to express our flexural parameters independently from stem diameter. Reducing water content in stems did not alter stem flexural strength, flexibility, or stiffness (Figure 6, C and D and Supplemental Figure S8), thereby showing that stem mechanical properties do not depend on water content but on cell walls. We then used two Arabidopsis mutants altered in their lignin G_CHO_ to G_CHOH_ contents to see how 7- to 8-week-old stem mechanical properties were affected. The mutants consisted of *fah1* (defective in FERULIC ACID 5-HYDROXYLASE 1), highly reduced in S and instead accumulating G_CHOH_ (Meyer et al., 1998; Blaschek et al., 2020b; Yamamoto et al., 2020), and *cad4 cad5* enriched in total and terminal G_CHO_ (Sibout et al., 2005; Blaschek et al., 2020a; Yamamoto et al., 2020). The *fah1* and *cad4 cad5* have similar stem growth and xylem organization as WT plants, only differing in lignin levels and composition (Jourdes et al., 2007; Thévenin et al., 2011; Vanholme et al., 2012). These biochemical changes differentially affected the TEs in whole stems of *fah1* and *cad4 cad5* compared to WT plants: lignin levels were slightly reduced but S/G, G_CHO_/G_CHOH_, and terminal/total G_CHO_ were largely altered (Figure 6E). We also observed similar biochemical changes in lignin at the whole stem level (Supplemental Figure S8). Flexural strength and stiffness were significantly different between genotypes, with each mutant being distinct from WT, and affected differently each stem segments (Figure 6, F and G). For the apical and middle segments, these changes included an ∼2-fold increase in stiffness when increasing G_CHOH_ in the *fah1* mutant and a ∼2-fold decrease when increasing G_CHO_ in the *cad4 cad5* mutant compared to WT (Figure 6F and Supplemental Figure S7). A similar reduction in stiffness due to increase of G_CHO_ in wood lignin had also been reported in poplar stems knocked down for *CAD* transcript levels (Özparpucu et al., 2017, 2018), revealing a conserved effect of G_CHO_ enrichment in lignin for herbaceous and woody species. By contrast, flexibility was unaltered between *fah1* and WT plants but increased by ∼3-fold in *cad4 cad5* for basal and middle stem segments and up to ∼4-fold for the apical segments (Figure 6G). Our results show that cell wall composition and more specifically lignin chemistry, independently of the developmental stages, alter stem biochemical properties. Our results moreover highlighted that the terminal aliphatic function of G residues in TEs had opposite effects on the cell wall biomechanics, increasing stiffness for alcohols compared to enhancing flexibility for aldehydes. Altogether, our results showed the importance of regulating G residues with specific aliphatic terminal functions to differentially modulate cell wall biomechanics, thus providing a mechanistic function behind the diversity of lignin residues.

### Increased TE cell wall flexibility due to coniferaldehyde in lignin enables plants to better recover from drought

**Figure 7:**
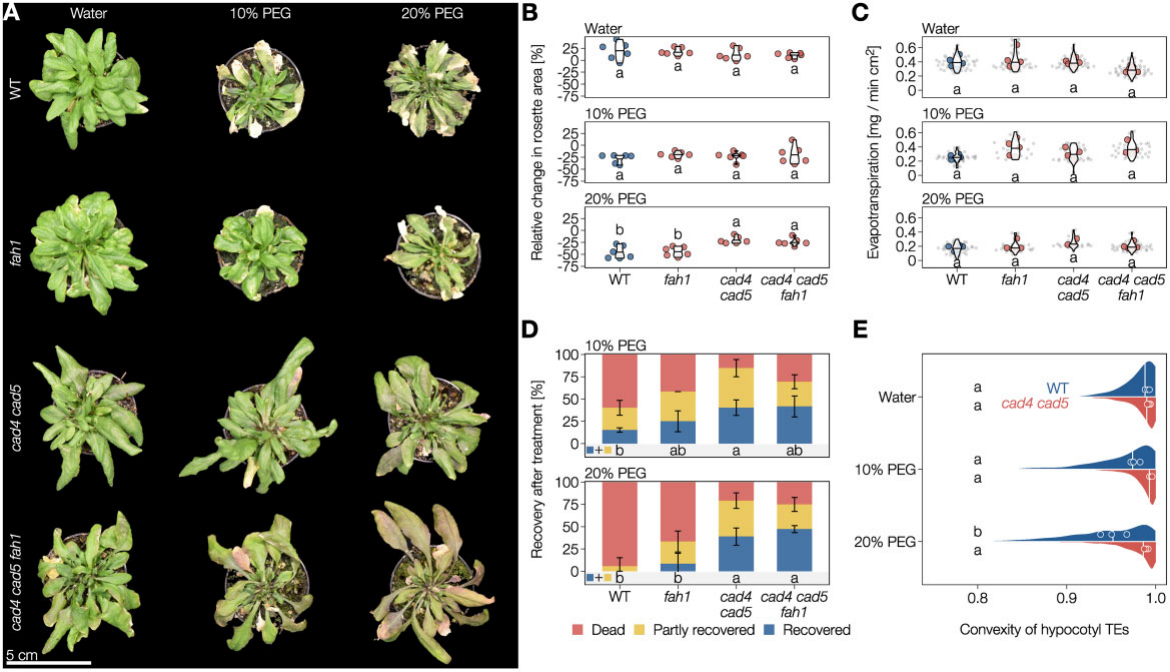
Coniferaldehyde-induced flexibility of TE lignin improves plant resistance and/or recovery from extreme Ψ differentials. A Top view of 4- to 5-week-old Arabidopsis WT, S-depleted *fah1*, G_CHO_-overaccumulating *cad4 cad5*, and S-depleted and G_CHO_-overaccumulating *cad4 cad5 fah1* mutant plants after being irrigated with water, 10% PEG6000 or 20% PEG6000 for 3 d. B, Relative change in projected rosette leaf area after 3 d of treatment with water, 10% PEG or 20% PEG, followed by 3 d recovery in water. Different lowercase letters indicate significant differences according to a Tukey-HSD test (per panel; α  =  0.05); *n *=* *6 plants per genotype and treatment. C, Evapotranspiration rates (normalized to the projected leaf area right before beginning of treatment) after irrigation with water, 10% PEG or 20% PEG for 3 d. Small gray dots represent individual measurements, larger colored dots represent the average per plant. Different lowercase letters indicate significant differences according to a Tukey-HSD test (per panel; α  =  0.05); *n *=* *3–6 plants per genotype and treatment. D, Proportion of plants that did not, partly or fully recover from treatment (with water, 10% PEG or 20% PEG for 3 d) after a 3-d recovery period in water-saturated soil. Different lowercase letters indicate significant differences in the proportions of plants that at least partly recovered according to a Tukey-HSD test (per panel; α  =  0.05); *n *=* *12–20 plants per genotype and treatment from two to three independent experiments. E, TE collapse in hypocotyls after PEG treatment and subsequent recovery in water. The distribution and median lines represent all measured TEs, median convexity for each individual plant is indicated by points. Different lowercase letters indicate significant differences between genotypes and treatments according to a Tukey-HSD test (α = 0.05); *n* = 3 individual plants per genotype and treatment.

As optimal vascular conduction is enabled by circular uncollapsed TEs (Zhang et al., 2016), we then evaluated how changes in biomechanics of TE cell walls affected their conductive function under drought conditions. More precisely, we estimated how changing TE biomechanics, either by increasing G_CHO_ or by increasing G_CHOH_ with less S in lignin, would affect sap conduction in response to normal watering or simulated drought using the osmoticum polyethylene glycol PEG6000 (Osmolovskaya et al., 2018). To ensure that the *cad4 cad5* and *fah1* mutants did not affect the lignin implicated for the endodermis function in water absorption/conduction, we monitored the apoplastic barrier capacity of *cad4 cad5*, *fah1*, and WT plants using propidium iodine staining of young seedlings (Lee et al., 2013). We observed no differences in apoplastic barriers between the *cad4 cad5*, *fah1* mutants, and WT plants (Supplemental Figure S9). Under normal conditions, 4- to 5-week-old WT plant rosettes had evapotranspiration rates of 0.4 mg water loss per min per leaf area, which decreased significantly by ∼50% under simulated drought when watered with 10% (w/v) and 20% (w/v) PEG6000 solution for 72 h (Figure 7, A–C and Supplemental Figure S10). The lowered evapotranspiration confirmed the normal response of plants to drought, essentially due to stomatal closure (Martin-StPaul et al., 2017). Leaf wilting and chlorosis were visible under drought and accentuated by the level of PEG6000 treatment (Figure 7, A and B). Recovery experiments by transferring plants to normal watering for 72 h showed that plants previously exposed to 20% PEG6000 did not recover, whereas only 15% of plants treated with 10% PEG6000 fully recovered (Figure 7D). Analysis of hypocotyl vasculature after re-watering showed a significant reduction in convexity increasing with PEG6000 concentrations (Figure 7E). This result indicated that drought in WT plants causes such strong inward collapse that TEs are unable to recover/restore their original shape after re-watering. In the *fah1* mutant, evapotranspiration rates also gradually decreased with increasing PEG6000 levels, producing plants with leaf wilting and some chlorosis but faring slightly better during recovery than the WT plants (Figure 7, A–D). The large reduction of S residues, or its compensatory increase of G_CHOH_, thus slightly benefited the plant capacity to recover from drought. In the *cad4 cad5* mutant, evapotranspiration rates also gradually decreased with increasing PEG6000 levels (Figure 7, A–D and Supplemental Figure S10). However, the mutant plants showed less wilting and chlorosis than the WT, resembling untreated plants (Figure 7, A and B). Recovery experiments after return to normal watering showed that ∼40% of *cad4 cad5* plants fully recover after both 10% and 20% PEG6000 treatments (Figure 7D). Analysis of hypocotyl vasculature in *cad4 cad5* plants after re-watering showed an apparent but nonsignificant collapse of TEs with PEG6000 levels (Figure 7E). Last, *cad4 cad5 fah1* plants showed evapotranspiration rates, leaf morphology, and recovery to drought similar to those of *cad4 cad5* (Figure 7, A–D), thus confirming that G_CHO_ independently of S residues affects the plant capacity to recover from drought. Overall, our results showed that the increased flexibility of TE cell walls, due to higher levels of G_CHO_ residues incorporation in lignin, either reduced TE capacity for irreversible inward collapse and/or increased TE capacity to recover their initial shape, thus enabling plants to better resist drought. We therefore show that lignin composition in the TE cell walls directly influences their hydraulic properties and capacity to sustain and/or recover from extreme environmental changes.

### Increasing coniferaldehyde residues in lignin changes molecular conformations, torsions and reduces stiffness compared to coniferyl alcohol residues

To understand how G_CHO_ and G_CHOH_ residues differently contributed to lignin biomechanics in TEs at the molecular level, we performed molecular dynamic simulations of lignin oligomers differing in their aliphatic parts. As G_CHO_ and G_CHOH_ residues are mostly interlinked with β–*O*–4 ether linkages *in planta* (Yamamoto et al., 2020), we designed β–*O*–4 interlinked homomeric heptamers of G_CHO_ and G_CHOH_ (with or without αC–OH) according to previous characterization studies (Grabber et al., 1998; Jourdes et al., 2007; Holmgren et al., 2009; Dima et al., 2015). Such lignin oligomers composed of only G_CHO_, G_CHOH,_ or S_CHOH_ have been chemically synthesized in vitro (Ito et al., 2002; Önnerud et al., 2002; Holmgren et al., 2009; Tobimatsu et al., 2010; Notley and Norgren, 2012; Koshiba et al., 2013; Blaschek et al., 2020a). Molecular dynamic simulations showed that oligomers of G_CHO_, which maintain an unsaturation in β–*O*–4 linkages (Grabber et al., 1998; Jourdes et al., 2007; Holmgren et al., 2009), greatly reduce the rotation of the _α_C–_β_C torsions but not the rotation around _β_C–O or C_6_–O–_α_C compared to G_CHOH_ with or without αC–OH (Supplemental Figure S11, A and B). Differences in molecular volume and density showed slightly larger and denser polymers for G_CHOH_, independently of αC–OH, compared to G_CHO_ (Table 1). The conformation of G_CHOH_ oligomers, independently of the αC–OH, was however substantially more compact with significantly smaller radius of end-to-end distance (Re-e) and of gyration (Rg) than the more extended conformations for G_CHO_ oligomers (Table 1). These findings showed the important influence of different terminal functions in the aliphatic chain of lignin residues, leading to polymers with very different conformations and thus affecting their capacity to fold and pack in the available space between the polysaccharides of plant cell walls during postmortem lignification (Supplemental Figure S11, C–E). Our results showed that increases in G_CHO_ would reduce the capacity of lignin polymers to compactly fold in these free cell wall spaces compared to G_CHOH_. This difference in compactibility could thus help explain why increases in G_CHO_ lead to lower lignin concentration in TE cell walls (Supplemental Figure S8G). We then performed molecular dynamic simulations of these different polymers under stress (external pressure) to evaluate the cumulative mechanical properties of 100 lignin oligomers made either of G_CHO_ or G_CHOH_ residues, with or without αC–OH. We observed significant increases in Young modulus (determining the relative stiffness to elasticity of any material), independently of αC–OH, for G_CHOH_ compared to G_CHO_ (Table 1). To define the influence of the aromatic part, we also performed molecular dynamic simulations on β–*O*–4 interlinked homomeric heptamers of S_CHOH_ with αC–OH. Compactibility and stiffness under stress of S_CHOH_ oligomers were similar to G_CHOH_ with αC–OH (Table 1 and Supplemental Figure S11). These results showed that changing the terminal aliphatic function of lignin residues directly alters the biomechanics of each polymer. Our results thus confirmed the differences in stiffness and flexibility observed in TEs and whole plants when modulating lignin chemistry (Figures 4–6). Our molecular dynamic simulation analyses showed that the modulation of G residue terminal aliphatic functions directly controls lignin biomechanics.

**Table 1:** Properties of lignin heptamers computed from molecular dynamics simulations

Oligomer	Volume	Density	Re-e	σ_(Re-e)_	Rg	σ_(Rg)_	Young modulus
	(Å^3^)	(g/cm^3^)	(Å)	(Å)	(Å)	(Å)	(MPa)
G_CHO_	1,690	1.21	19.6	8.5	9.5	1.7	1,400 ± 200
G_CHOH_	1,740	1.205	13.6	5.9	7.25	0.75	2,000 ± 200
G_CHOH_-αC-OH	1,790	1.25	14.1	5.4	7.8	1.2	1,900 ± 200
S_CHOH_-αC-OH	2,110	1.23	15.2	6.6	7.9	1.2	2,100 ± 200

Re-e is the root mean square of the end-to-end distance and σ_(Re-e)_ is its variance; Rg is the average radius of gyration and σ_(Rg)_ is its variance.

## Discussion

TEs belong to the few cell types that fulfill their function only after their death, which greatly limits their capacity to adapt to changing environmental or developmental constraints. Accordingly, previous studies have assumed that functional TEs were inert and had no adaptive capacity. We however showed here that TEs can still adapt after death by accumulating lignin postmortem in their SCWs (Figures 1 and 5 and Supplemental Figure S6). This process occurred in TEs from both annual and perennial species (Figures 1 and 5 and Supplemental Figure S6), thereby setting postmortem lignification as a conserved mechanism of TEs made to reinforce their conducting roles once hollowed out by cell death in many vascular plants. We moreover showed that TE postmortem lignification not only increased total lignin concentration but also changed its composition as the TE matured (Supplemental Figure S6). We further confirmed that TE cell walls were specifically enriched in G residues like in other tracheophytes (Pesquet et al., 2019), and showed that each TE morphotype has a distinct lignin composition that cannot be limited to S/G ratio (Figures 3 and 5). This specific cell wall lignin composition for each TE morphotype is essential to prevent inward collapse for an optimal hydraulic conductivity, and the incorporation of wrong residues compromises the biomechanics of most TE morphotypes. This result complemented recent observations that showed the collapse of MX in Arabidopsis when their S content was genetically increased (Sakamoto et al., 2020). This morphotype-specific resolution of lignin highlights the benefits of using in situ methods capable of measuring biochemical and biomechanical aspects at the single cell and even cell wall levels, such as SEM-EDS, AFM, and Raman microspectroscopy (Figures 1–6). Unlike biochemical methods using ball-milling and grinding, these in situ quantitative imaging methods avoid averaging errors and compound effects between cell types at different maturation stages when analyzing whole plant organs. However, the direct analysis of lignin in situ using Raman spectroscopy does not enable a reliable determination of H residue proportion or the different aliphatic functions of S residues (Blaschek et al., 2020b). We show that the different aliphatic terminal function of G residues, such as alcohol or aldehyde, specifically accumulated in different concentrations between TE morphotypes (Figures 4 and 5) during TE postmortem maturation, with a later deposition of G_CHO_ compared to G_CHOH_ (Supplemental Figure S6; Kutscha and Gray, 1972; Blaschek et al., 2020a). This difference in temporal accumulation of G_CHO_ compared to G_CHOH_ appears essential for the biomechanical properties of lignin in TEs, as the untimely incorporation of G_CHO_ hinders TE capacity to resist collapse (Figure 5). Specific control of G_CHOH_ and G_CHO_ accumulation in lignin had previously been shown between wood cell types and cell wall layers in multiple plant species (Peng and Westermark, 1997; Kim et al., 2015; Zheng et al., 2016; Blaschek et al., 2020a), during postmortem maturation (Blaschek et al., 2020a) and in response to environmental conditions (Euring et al., 2012; Nakagawa et al., 2012; Camargo et al., 2014). We showed that each TE morphotype required a specific G_CHO_/G_CHOH_ compositional ratio to fine-tune the stiffness to flexibility of their cell walls in single cells as well as in whole stems (Figures 2–6 and Supplemental Figure S8). The adjustment of stiffness to flexibility of ether-linked G residues only depended on the terminal aliphatic function rather than on aromatic ring substitutions as shown by our molecular dynamic simulations (Table 1 and Supplemental Figure S11). From an evolutionary perspective, the adjustment of lignin biomechanics by changing the residue aliphatic functions may have arisen before changing aromatic ring substitutions, as conifers respond to drought with TE collapse and present large amounts of G_CHO_ but are devoid of any S residues (Cochard et al., 2004; Wagner et al., 2012; Blaschek et al., 2020a). As TE dimensions and cellular surroundings greatly vary between plant species, our results moreover show that each plant species fine-tunes the G_CHO_/G_CHOH_ with other changes in lignin chemistry for their distinct TEs (Figures 3–5 and Supplemental Figures S4–6). In addition to the role of lignin chemistry to support TE function, our results confirmed that the vascular tissue organization (TE neighboring others TEs) affected TE biomechanics and hydraulics but only for specific morphotypes (Figures 4 and 5; Lens et al., 2011; Martínez-Vilalta et al., 2012). Changes in cell wall stiffness to flexibility at the TE level might represent a mechanism to adapt TE biomechanics to environments where water availability varies brutally, thus enabling TEs to regain their original shape after collapse to restore plant sap conduction. We suggest that the trade-off between stiffness and flexibility of TEs is regulated by controlling lignin composition. Crucially, this regulation continues after TE cell death, demonstrating that TEs are not an inert arrangement of pipes, but rather a dynamic system that can continuously adjust its properties through postmortem lignification. The exact identity and function of the different components enabling TE postmortem lignification such as the living cooperating cell types, the metabolites used, and molecular actors regulating lignin formation through biosynthesis, transport, and/or polymerization still need to be identified. Our study therefore establishes that the proportions of the different lignin residues, varying in both aromatic and aliphatic substitutions, are specifically regulated in each cell types and along their maturation to enable specific cellular functions.

## Materials and methods

### Inducible pluripotent cell suspension cultures (iPSCs)

Arabidopsis (*A. thaliana*) iPSCs were produced and induced to differentiate into isolated TEs as previously described (Pesquet et al., 2010; Ménard et al., 2017). Cell suspensions were induced by adding phytohormones to 30 mg mL^−1^ of 10-d-old cells (fresh weight) in 1× Murashige and Skoog (MS) medium (Duchefa, M0222.0025) at pH 6.0 with 10 µM of morpholino-ethanesulfonate (Sigma-Aldrich, M8250) and 3% (w/v) sucrose. Xylogenic induction was triggered by adding 6 µg mL^−1^ α-naphthaleneacetic acid (Sigma Aldrich, N0640), 1 µg mL^−1^ 6-benzyl-aminopurine (Sigma Aldrich, B3408), and 4 µM 24-epibrassinolide (Sigma Aldrich, E1641). The inhibition of lignin monomer biosynthesis was performed by adding 12.5 µM of PA (Sigma-Aldrich, P49805) at the time of phytohormone induction of TE differentiation in suspension cultures as previously described (Pesquet et al., 2013; Van de Wouwer et al. 2016; Decou et al., 2017).

### Plant material

Arabidopsis and hybrid poplar (*Populus tremula×tremuloides*) plants were grown in controlled growth chambers under a 16-h light/8-h dark photoperiod with 150 µmol m^−2^ s^−1^ illumination (using Aura T5 Eco Saver Long Life HO light tubes; AuraLight, Sweden) and a 22°C/18°C temperature cycle in 60% humidity. Arabidopsis mutants in the Columbia (Col-0) background used included *ccoaomt1* (SALK_151507; Kai et al., 2008), *fah1* (EMS mutant; Meyer et al., 1998), *omt1* (SALK_135290; Tohge et al., 2007), *4cl1-1* (SALK_142526; Van Acker et al., 2013), *4cl2-4* (SALK_110197; Li et al., 2015), *4cl1 4cl2* (Blaschek et al., 2020a), *ccr1-3* (SALK_123-689; Mir Derikvand et al., 2008), *cad4* (SAIL_1265_A06; Lee et al., 2017), *cad5* (SAIL_776_B06; Lee et al., 2017), *cad4 cad5* (Blaschek et al., 2020a), and *cad4 cad5 fah1*. All mutants were checked for homozygosity using PCR genotyping as previously described by Blaschek et al. (2020a). *Populus tremula×tremuloides* hybrid poplars clone T89 were transformed as described by Nilsson et al. (1992) with RNAi constructs targeting either *CINNAMATE-4-HYDROXYLASE* (Potri.013G157900; Bjurhager et al., 2010) or *CINNAMOYL-COA REDUCTASE* (Potri.003G181400) selected for best reduced gene expression (Escamez et al., 2017). Poplar plants were first propagated in vitro and grown for 2 months and then acclimated to soil for 7–12 months in conditions identical to those described above.

### Atomic force microscopy

AFM imaging was performed on cell samples semi-dried for less than 1 h using a Dimension Icon AFM (Bruker, Nanoscope controller, Santa Barbara, CA, USA). The measurement was conducted under Peak-Force QNM mode in air condition with a TESPA-V2 probe (Bruker). The force set-point was 0.15 V. The height, peak-force error, Derjaguin–Muller–Toporov (DMT) modulus, adhesion, and deformation images were recorded after calibrating the probes on mica. The images were processed by NanoScope Analysis 1.5 software (Bruker) and quantification was performed using ImageJ distribution Fiji (Schindelin et al., 2012).

### SEM coupled with EDS

Water-washed cell samples without fixation were dispersed and sedimented on glass coverslips, then either (1) dehydrated through a graded ethanol series and critical-point dried (CPD) using a Leica EM CPD300 critical-point dryer (dehydrated in series of ethanol gradient from 70%, 80%, 90%, 95% to 2 times in 100% (v/v) for 10 min each, critically point dried for ∼2 h to exchange ethanol to liquid carbon dioxide for 16 times at 35°C and 74 bar pressure), or (2) subjected to air drying (dried for 24 h at 20°C and 1 bar atmospheric pressure), and finally coated with 5 nm chromium using Quorum Technologies Q150T ES metal coater. The morphology of samples was analyzed by field-emission SEM (Carl Zeiss Merlin) using an in-lens secondary electron detector at accelerating voltage of 4 kV and probe current of 100 pA. Elemental composition measurements were performed using an energy-dispersive X-ray spectrometer (EDS; Oxford Instruments X-Max 80 mm^2^) at an accelerating voltage of 10 kV and a probe current of 300 pA, where the elemental composition percentage is an average of multiple line and point analyses.

### Histological preparation and analyses

Eight-week-old inflorescence stem bases or 4- to 5-week-old hypocotyls were cleared in 70% (v/v) ethanol, rinsed in water, and embedded in 10% (w/v) agarose prior to sectioning to 50 µm with a VT1000S vibratome (Leica, Sweden). TE cell wall autofluorescence was acquired using laser scanning confocal microscopy (LSM800 Zeiss, Germany) with excitation at 405 nm and emission collected from 420 nm to 650 nm (Decou et al., 2017). Semi-quantitative Raman microspectroscopy was performed as described by Blaschek et al. (2020b) on the different TE/vessel types using a confocal Raman microscope (RAMANplus, Nanophoton, Japan and LabRAM HR 800, Horiba, France) with a 532 nm laser. Averaged spectra were obtained from three to seven cell walls per TE morphotype and per plant, with one to three plants per genotype for Arabidopsis, and from 17 to 71 cell walls per TE morphotype and per plant, with two to six plants for poplar. Quantitative Wiesner test was performed as described by Blaschek et al. (2020a) using an Olympus BX60 brightfield microscope equipped with an Olympus UPFLN 40X objective (NA 0.75), and an Olympus XC30 CCD color camera. TE morphological features (distance from cambium, lumen area, perimeter, circularity, neighboring cell types) were measured from microscopy images using ImageJ distribution Fiji (Schindelin et al., 2012). TE circularity was determined as 4π (area/perimeter^2^), and TE convexity as area/area of convex hull. Fiji macros are available at https://github.com/leonardblaschek/fiji.

### Three-point flexural test

The stiffness and strength of stems were assessed using three-point flexural tests with an Instron 5966 universal testing machine (Instron, USA) equipped with a 100 N load cell in a humidity- and temperature-controlled room (50% relative humidity at 23°C). Stem segments of 4–5 cm in length from 25–35 or 35–45 cm-long stems of 5–6 or 7–8 week-old plants were placed on two supporting pins that were separated by an average span-to-diameter ratio of 38–39 ± 4. Treatment to alter stem water content included incubation for several hours prior to bending in pure distilled water or 1 M sorbitol solution (Sigma, S1876); all other measurements were performed in air. After manually lowering the loading pin until just becoming in contact with the sample, the probe was lowered automatically at a constant displacement rate of 2 mm min^−1^ until a final displacement of 7 mm (Supplemental Movie S1). The flexural strength σ_max_ (MPa) was calculated as the maximum flexural stress using Equation 1.

(1) $$\sigma_{\max}=\frac{8F_{\max}L}{\pi d^{3}}$$

In this equation, *F*_max_ (N) is the maximum force the specimen can withstand before irreversible breaking, L (mm) is the span length between the supporting pins, and d (mm) is the diameter of the circular cross-section of the specimen that was determined using optical microscopy imaging. The flexural stiffness E (MPa) was calculated from the slope of the initial linear part of the flexural stress-strain curve using Equation 2.

(2) $$E=\frac{4FL^{3}}{3\pi Dd^{4}}$$

In this equation, D (mm) is the maximum deflection of the center of the stem. The flexibility of stems was defined as the strain at maximum stress, *i.e.* the amount of deformation a stem can endure before irreversibly breaking.

### Evapotranspiration and simulated drought

Simulated drought treatments were conducted by watering plants with 0%, 10%, or 20% (w/v) polyethylene glycol (PEG) 6000 (Sigma-Aldrich, 8.07491) mixed in tap water for 72 h in growing conditions (150 µE light, 25°C, 60% relative humidity). Evapotranspiration was measured using mass difference over 20 min on a LA-124i microbalance (VWR) directly connected to a computer and monitored using the i-Weight software (VWR). Plant recovery was accomplished by placing potted plants directly in water for 72 h, and recovery was scored as restoration of leaf flaccidity (partial recovery) together with bolting (full recovery). Light intensity, temperature, and relative humidity were constantly monitored during the course of the measurements (Supplemental Figure S10). Images of the rosettes were acquired with Nikon D750 camera equipped with a 50-mm F1.4 DG HSM lens. Image segmentation and rosette area measurements were performed in Fiji (Schindelin et al., 2012). Hypocotyl vessel collapse in normal and simulated drought conditions was estimated as described above.

### Apoplastic connectivity staining

Arabidopsis seeds were surface sterilized (2 min in 70% [v/v] ethanol followed by 5 min in 5% [v/v] bleach) and stratified in water for 2 d at 4°C. Seeds were plated onto half-strength MS medium, pH 5.7 with 0.8% (w/v) agar, and placed in a growth chamber under a 16-h light/8-h dark photoperiod with 150 µmol m^−2^ s^−1^ illumination (Aura T5 Eco Saver Long Life HO light tubes; AuraLight, Sweden) and 22°C/18°C temperature cycle in 60% humidity. Seedlings were grown vertically for 4 d. Formation of the functional apoplastic barrier was analyzed using propidium iodide (PI; Sigma-Aldrich P4170) as described previously (Lee et al., 2013). Briefly, seedlings were stained in 15 µM PI in deionized water for 10 min, rinsed twice in tap water, mounted in tap water between glass slide and cover slip, and imaged using a Zeiss LSM 780 confocal microscope equipped with a 20× objective. PI staining of the apoplastic space was excited with a 488-nm laser and visualized by long pass emission at >500 nm. Tiles were stitched and analyzed in Fiji (Preibisch et al., 2009; Schindelin et al., 2012). For quantification, cells were counted from onset of elongation (defined as cells being more than twice as long as they are wide) to the absence of any stain from the vascular cylinder.

### Lignin biochemical analysis

Lignin concentration in the cell wall was determined after cell wall isolation according to Yamamoto et al. (2020) and thioglycolic acid lignin derivatization as described by Suzuki et al. (2009) on isolated extractive-free cell walls. Absorbance was measured at 280 nm and calibrated using a regression curve obtained using different quantities of alkaline spruce lignin (Sigma Aldrich, 471003). Pyrolysis-gas chromatography/mass spectrometry (pyrolysis-GC/MS) was used to measure S/G and G_CHO_/G_CHOH_ according to Gerber et al. (2012) on 60 µg (±10 µg) of 8-week-old stem samples. Thioacidolysis-GC/MS-FID was used to determine the terminal/total positional ratio of β-*O-*4 linked G_CHO_ residues on 5 mg (±1 mg) of isolated cell wall from 8-week-old stem samples as described by Yamamoto et al. (2020).

### Data analyses and structural equation modeling

Data analysis and visualization were performed in R (v4.0.4), using the “tidyverse” collection of packages (v1.3.0; Wickham et al., 2019). Parametric and nonparametric multiple comparisons were done using the “tukeygrps” package (https://github.com/leonardblaschek/tukeygrps), integrating functions from the “stats,” “dunn.test,” and “multcompView” packages. The structural equation models were built using the “piecewiseSEM” package (v2.1.0; Lefcheck, 2016). The included variables were measured for each individual TE, except for the Wiesner test intensity in Arabidopsis, for which the average per individual plant and TE type was used. The multiple linear regression models in the structural equation models were selected using a bidirectional stepwise optimization approach. The structural equation models containing the optimized multiple regressions then allowed us to integrate the information and ascertain that lignin directly affected inward collapse (i.e. convexity) and not general deformation (i.e. circularity). Interaction terms were excluded from the models in the main figures to ease interpretation. Interaction terms that would significantly improve the models were identified separately and visualized using the “interactions” R package (v1.1.1). Model fits and coefficients are summarized in Supplemental Tables S3 and S4. Data and statistical analyses are also provided in Supplemental Data Sets 1 and 2. R code used in this study is available at https://github.com/leonardblaschek/Rscripts/blob/master/irx_pub_figs.rmd.

### Molecular dynamic simulations

Molecular dynamics simulations were carried out on lignin heptamers made of either only G_CHOH_, with or without αC–OH, G_CHO_, or S_CHOH_ with αC–OH interlinked by β–*O*–4 ether linkages according to previous analyses (Grabber et al., 1998; Holmgren et al., 2009; Yamamoto et al., 2020). Molecular structures were designed with the Avogadro software (Hanwell et al., 2012) and processed using ACPYPE utility (Sousa da Silva and Vranken, 2012) to produce GROMACS (Abraham et al., 2015) topology files implementing Generalized Amber Force Field (GAFF; Wang et al., 2004). All simulations were carried out using GROMACS v.2020 software. First, 100 molecules of the selected type were placed randomly in a cubic box of size 20 nm in random orientations. Short energy minimization run was carried out to remove possible molecule overlap. This was followed by 1-ns run at 100 bar pressure to push the oligomers close to each other, then a 400-ns run at 1 bar pressure with isotropic Berendsen barostat (Berendsen et al., 1984), and then 400 ns with anisotropic barostat. The obtained system was considered at equilibrium and used for further analysis. Young modulus computations were carried out by applying pressure between 100 and –200 bar with a step of 50 bar, in each of three directions. At each pressure step, a 300-ns simulation was carried out, the last 100 ns were used to determine average box extension. The Young modulus was determined from the slope of strain–stress plot averaged in each of the three spatial directions. Simulations were repeated by starting from another random configuration and using the same protocol. Other relevant simulation parameters included: temperature control with Berendsen thermostat at T = 298°K and relaxation time 1 ps, time step 2 fs, constraint bonds to hydrogen atoms, Particle mesh Ewald summation of electrostatic interaction (Darden et al., 1993).

## Accession numbers

Hybrid poplar—CINNAMATE-4-HYDROXYLASE (C4H): Potri.013G157900/CINNAMOYL-COA REDUCTASE (CCR): Potri.003G181400.

Arabidopsis—4-COUMARATE-COA LIGASE 1 (4CL1): At1g51680/4-COUMARATE-COA LIGASE 2 (4CL2): At3g21240/CAFFEOYL-COA O-METHYLTRANSFERASE 1 (CCoAOMT1): At4g34050/FERULIC ACID-5-HYDROXYLASE 1 (F5H1): At4g36220/CAFFEIC ACID O-METHYLTRANSFERASE 1 (OMT1): At5g54160/CINNAMOYL-COA REDUCTASE 1 (CCR1): At1g15950/CINNAMYL ALCOHOL DEHYDROGENASE 4 (CAD4): At4g37980/CINNAMYL ALCOHOL DEHYDROGENASE 5 (CAD5): At4g37990.

## Supplemental data

The following materials are available in the online version of this article.

**Supplemental Figure S1.** Introduction to basic physiological concepts of water conduction in TEs.

**Supplemental Figure S2.** MX and PX TEs continuously lignify postmortem.

**Supplemental Figure S3.** In situ quantification of lignin chemistry by Raman microspectroscopy.

**Supplemental Figure S4.** TE morphology and lignin composition in 8-week-old Arabidopsis phenylpropanoid mutants.

**Supplemental Figure S5.** Effects of cell wall morphology and composition on convexity in Arabidopsis are interdependent.

**Supplemental Figure S6.** TE morphology and lignin composition in *Populus tremula*×*tremuloides* phenylpropanoid transgenic lines.

**Supplemental Figure S7.** Effects of cell wall morphology and composition on convexity in poplar are interdependent.

**Supplemental Figure S8.** Stem biomechanics and lignin composition in *fah1* and *cad4 cad5*.

**Supplemental Figure S9.** Apoplastic barrier of the endodermis is unaffected in *fah1* and *cad4 cad5.*

**Supplemental Figure S10.** Conditions and hypocotyl TE collapse during the drought experiment.

**Supplemental Figure S11.** Topology and mechanics of lignin oligomers depend on the C_3_ functional group.

**Supplemental Table S1.** Used nomenclature of lignin chemistry.

**Supplemental Table S2.** Insertional mutants and the targeted genes used in the present study, with gene name, locus number, number of paralog for each plant species, and previous references in which these plants were analyzed.

**Supplemental Table S3.** Test statistics on global goodness-of-fit (Fisher’s C) and directed separation (i.e. independence of variables) in the piecewise structural equation models (related to Figures 4 and 5).

**Supplemental Table S4.** Standardized and raw coefficients and their *P*-values in the piecewise structural equation models (related to Figures 4 and 5).

**Supplemental Data Set S1.** Summary of all data obtained in this study.

**Supplemental Data Set S2.** Summary of statistical analyses in this study.

## Supplementary Material

koac284_Supplementary_Data

## Notes

E.P. conceived the study. E.P. and D.M. designed the experiments. D.M., L.Bl., K.K., C.Z., H.S., N., C.C.L., A.L., Z.B., and E.P. performed the experiments. D.M., L.Bl., K.K., C.C.L., A.L., and E.P. analyzed the data. L.Be., A.M., Z.B., S.K., and E.P. ensured financial support and scientific expertise. E.P. wrote the article. All co-authors revised the manuscript.

The author responsible for distribution of materials integral to the findings presented in this article in accordance with the policy described in the Instructions for Authors (https://academic.oup.com/plcell) is: Edouard Pesquet (Edouard.pesquet@su.se).
