# Supplementary material for: Plant biomechanics and resilience to environmental changes are controlled by specific lignin chemistries in each vascular cell type and morphotype
Source: Plant Cell. 2022 Sep 21;34(12):4877–96. doi: 10.1093/plcell/koac284 (PMC9709985; doi:10.1093/plcell/koac284)
Supplement: koac284_Supplementary_Data [file koac284_supplementary_data.zip › Menard2022_supplement.pdf]

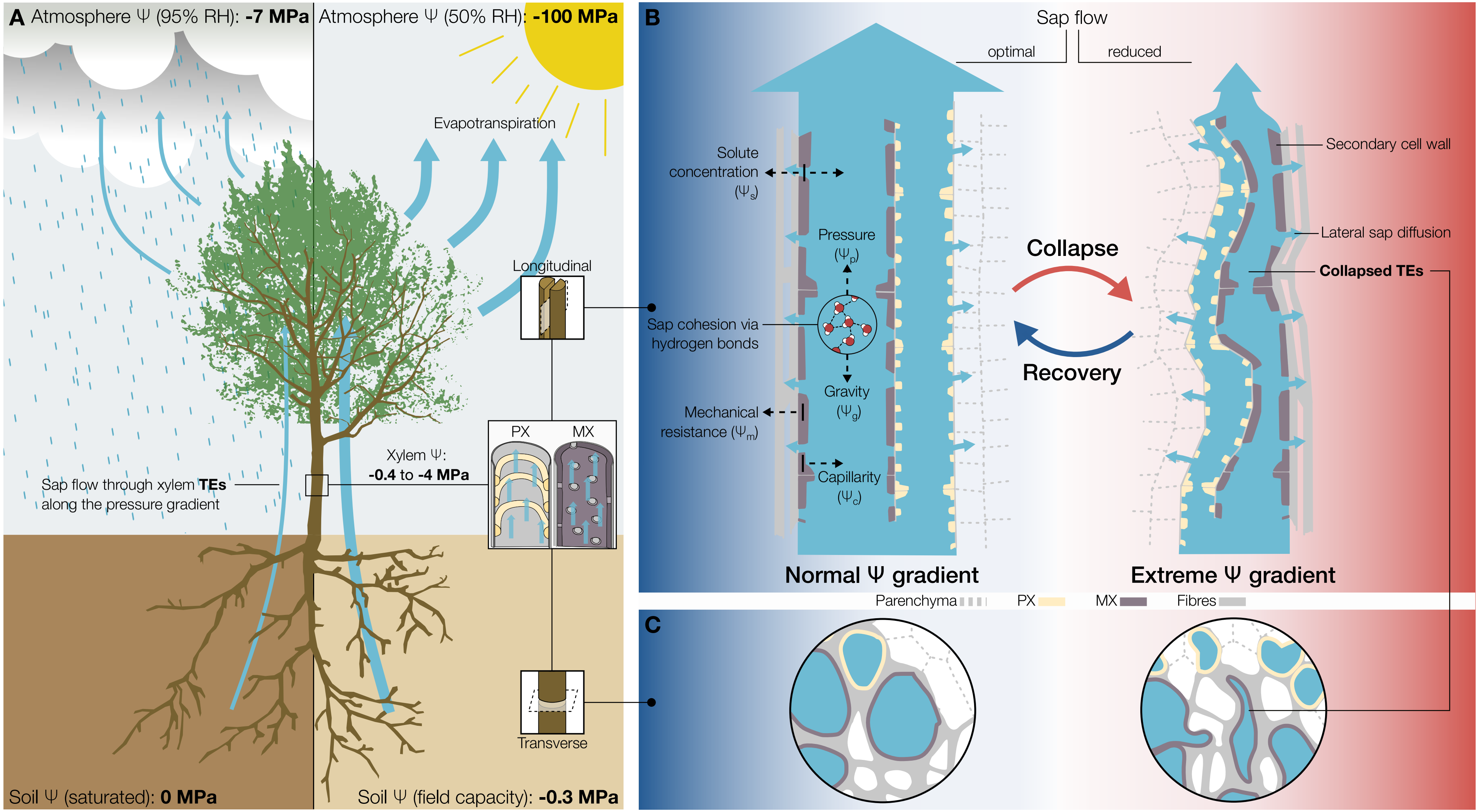

Supplemental figure S1 | Introduction to basic physiological concepts of water conduction in TEs (related to the introduction).
A The gradient of Ψ between soil, plant and atmosphere that drives water transport through TEs depends on environmental conditions. **B, C** Schematic longitudinal **(B)** and transverse **(C)** sections through the different TE morphotypes. The total Ψ is the sum of physical (Ψc, Ψg, Ψm, Ψp) and chemical (Ψs) pressures. Under normal conditions, TEs can withstand Ψ without inward collapse, but extreme environmental changes and the associated large gradient in Ψ leads TEs to collapse, which affects water flow. Once the conditions return to normal, collapsed TEs can regain their shape. MX, metaxylem; PX, protoxylem; RH, relative humidity; TE, tracheary element.

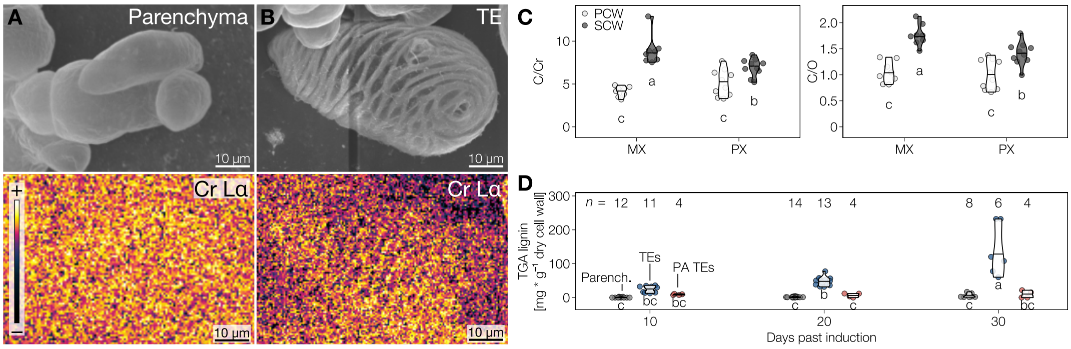

Supplemental figure S2 | MX and PX TEs continuously lignify *post-mortem* (related to figure 1).
A Scanning electron micrograph of isolated parenchyma cell in uninduced condition, prepared using critical-point drying (CPD), and its corresponding energy-dispersive X-ray spectroscopy (EDS) chromium (Cr) signal in color-coded intensity. **B** Scanning electron micrograph of isolated TE after 30 days in induced condition, prepared using CPD, and its corresponding EDS Cr signal in color-coded intensity. **C** C/Cr and C/O ratios in PX and MX TEs from the plateau phase of cell wall lignification (>30 d after induction); n = 8–10 individual cells per morphotype. **D** Lignin content of extractive-free cell walls from parenchyma cells, lignifying TEs and TEs treated with piperonylic acid (PA, 12.5 µM) determined using thioglycolic acid derivatization (TGA); n = 4–14 independent cultures per time point and treatment. Different lowercase letters indicate significant differences according to a Tukey-HSD test (per panel; α = 0.05).

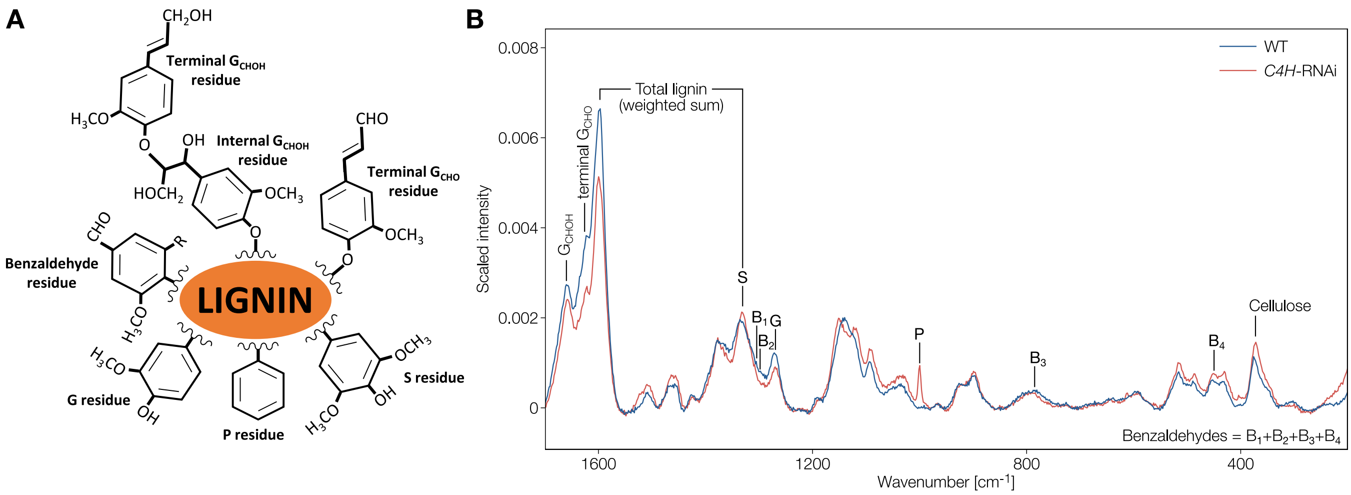

Supplemental figure S3 | *In situ* quantification of lignin chemistry by Raman microspectroscopy (related to figures 3–6).
A Lignin residues detected by Raman microspectroscopy (R = –H or $–OCH_3$). **B** Raman bands used for the quantification of different cell wall polymers and lignin residues, shown on the example of representative spectra of TEs from *Populus tremula×tremuloides* WT and *C4H*-RNAi plants. The scattering of P (styrene) residues at 1,000 cm^{-1} is described in Noda and Sala (2000).

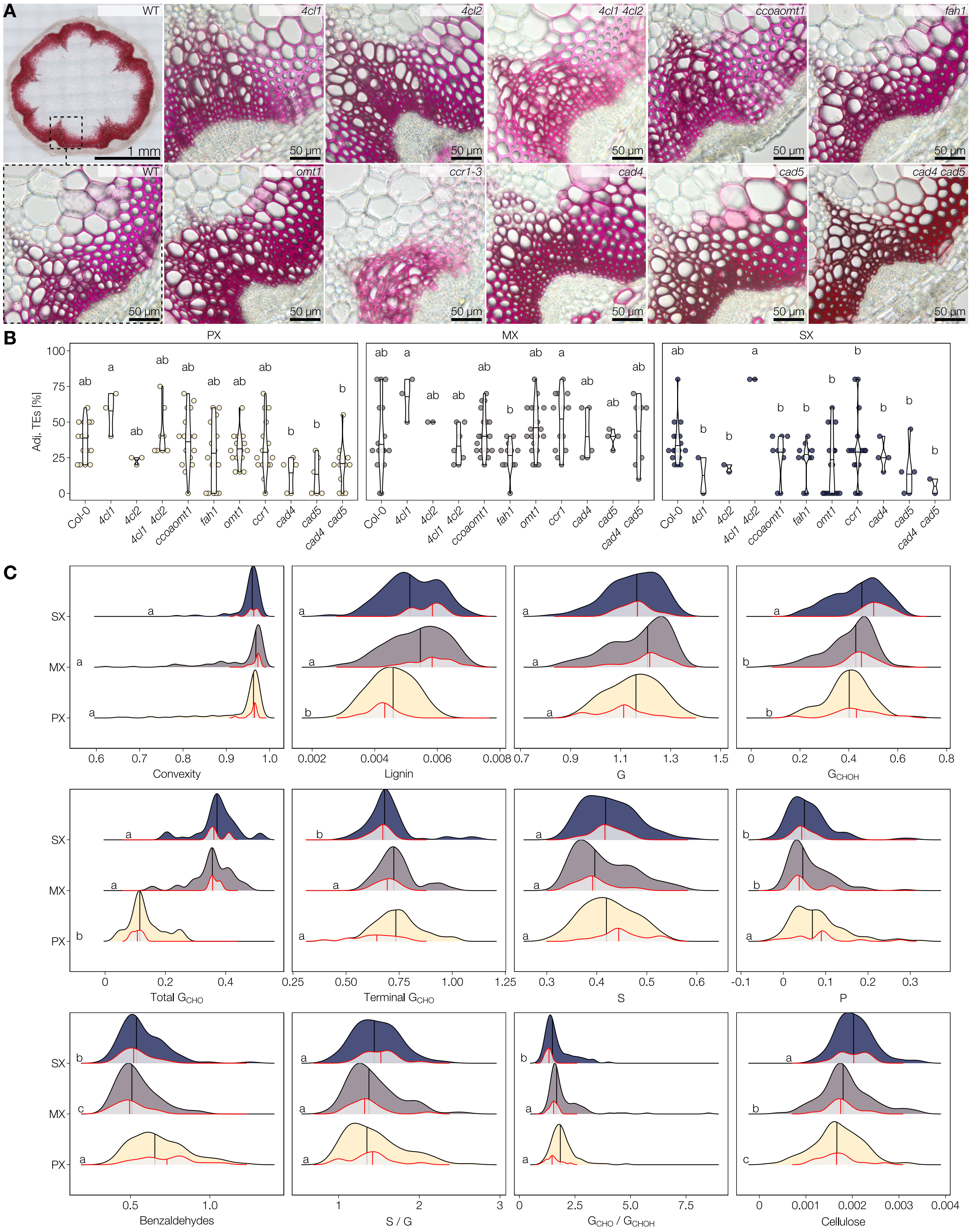

Supplemental figure S4 | TE morphology and lignin composition in eight-week-old Arabidopsis phenylpropanoid mutants (related to figure 4).

A Representative Wiesner-stained sections of the WT and the phenylpropanoid mutants used. **B** Relative proportion of cell types surrounding TE perimeters other than TEs in the different Arabidopsis genotypes comprising our dataset. Different lowercase letters indicate significant differences between genotypes according to a Tukey-HSD test (per panel; $\alpha = 0.05$). **C** Arabidopsis TE convexity and cell wall composition data used in the structural equation models (Figure 4 B–D). Variation across all genotypes (in blue/purple/yellow) overlayed with the variation in the WT (gray with red outline, scaled to 30%). Vertical lines represent the respective median values. Different lowercase letters indicate significant differences between TE morphotypes according to a Kruskal-Wallis test followed by Dunn's multiple comparison (per panel; $\alpha = 0.05$). All data used in the models is also available in Supplemental Data Set S1.

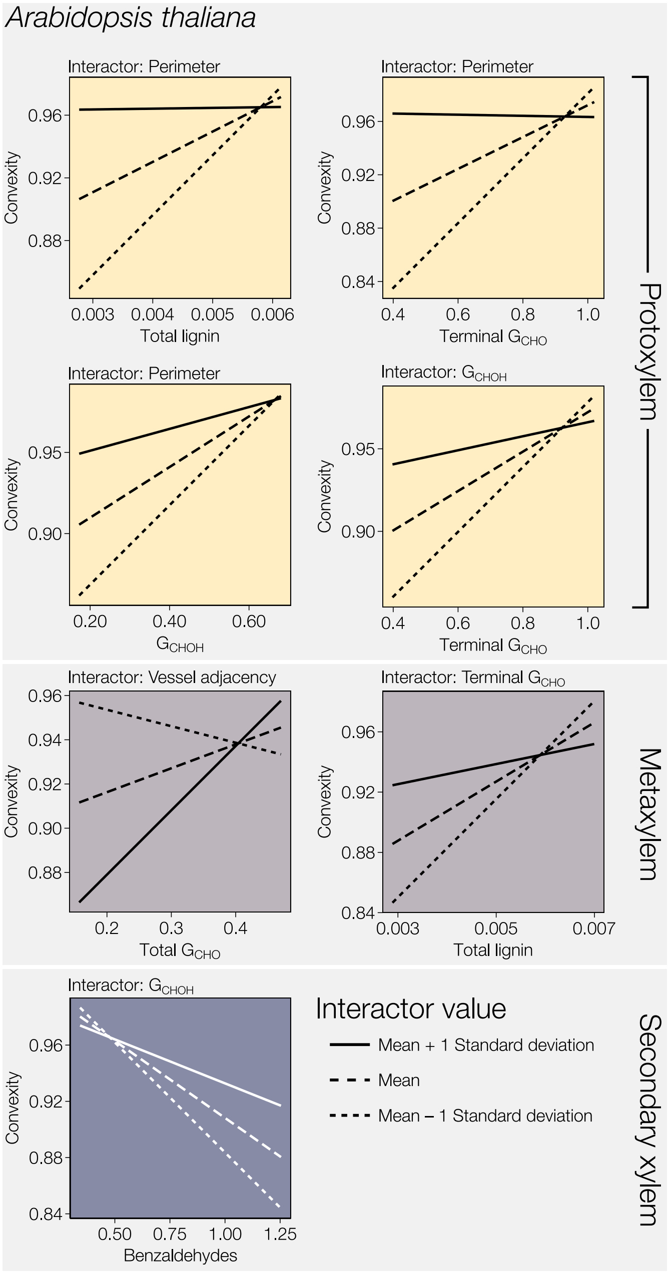

Supplemental figure S5 | Effects of cell wall morphology and composition on convexity in Arabidopsis are interdependent (related to figure 4).

Several two-way interactions between biochemical and morphological predictors significantly affected TE convexity. The lines represent effects of the respective variable on convexity when the interactor value was high (solid line), average (dashed line), or low (dotted line).

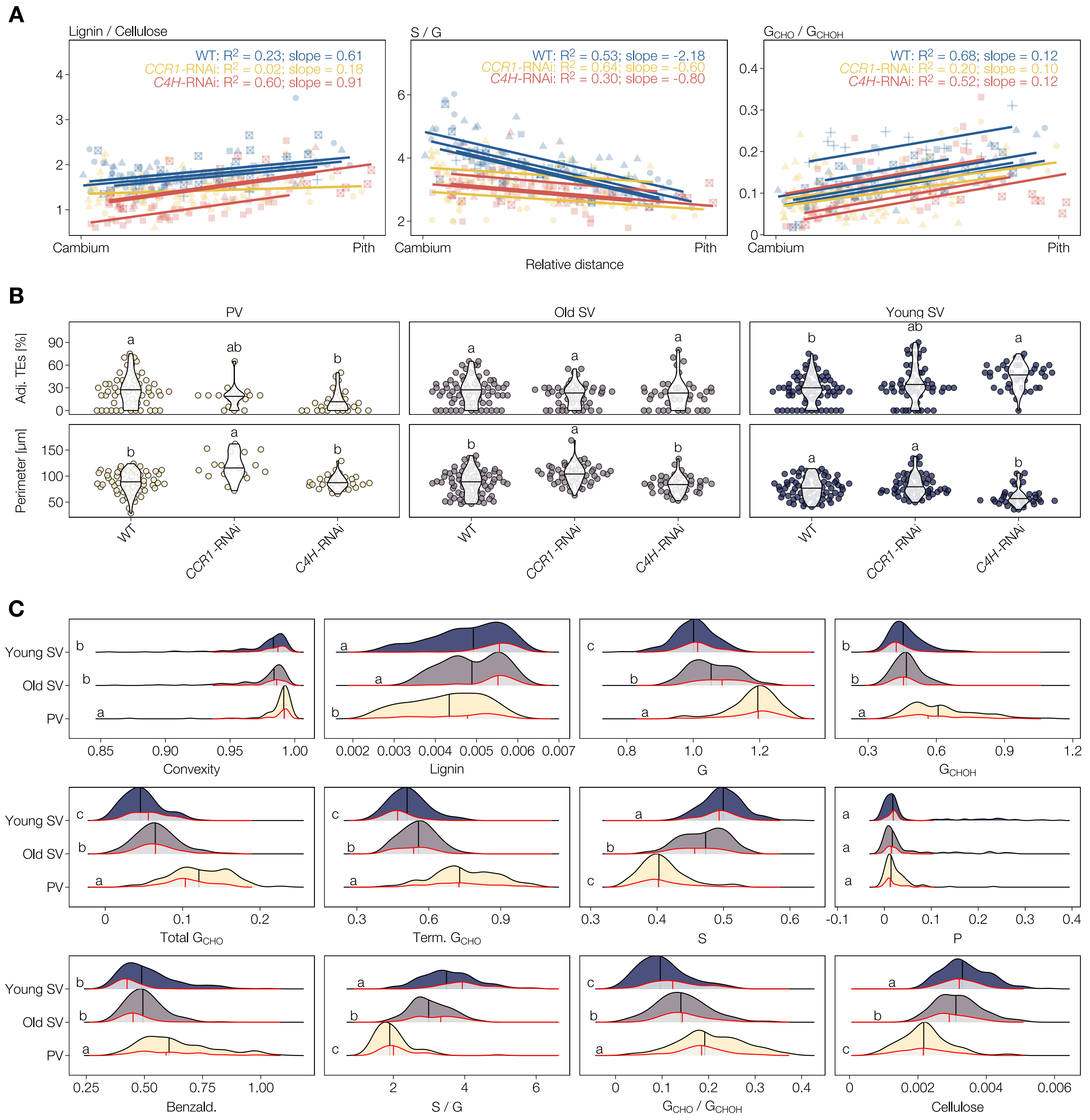

Supplemental figure S6 | TE morphology and lignin composition in *Populus tremula*×*tremuloides* phenylpropanoid transgenic lines (related to figure 5).

A *Post-mortem* lignification in *Populus tremula*×*tremuloides* TEs. The ratios of lignin to cellulose, S to G, and G_{CHO} to G_{CHOH} in secondary TEs of poplar stems changed with their distance to the cambium, *i.e.* their age. The lines, conditional R^2 values and slopes represent mixed linear models of the respective ratio against the distance from the cambium, allowing for different intercepts for each plant. **B** Relative proportion of TE perimeters surrounded by other TEs and TE perimeters in the different genotypes comprising our dataset. Different lowercase letters indicate significant differences between genotypes according to a Tukey-HSD test (per panel; $\alpha = 0.05$). **C** *Populus tremula*×*tremuloides* TE convexity and cell wall composition data used in the structural equation models (Figure 5 H–J). Variation across all genotypes (in blue/purple/yellow) overlayed with the variation in the WT (gray with red outline, scaled to 30%). Vertical lines represent the respective median values. Different lowercase letters indicate significant differences between TE morphotypes according to a Kruskal-Wallis test followed by Dunn's multiple comparison (per panel; $\alpha = 0.05$). All data used in the models is also available in Supplemental Data Set S1.

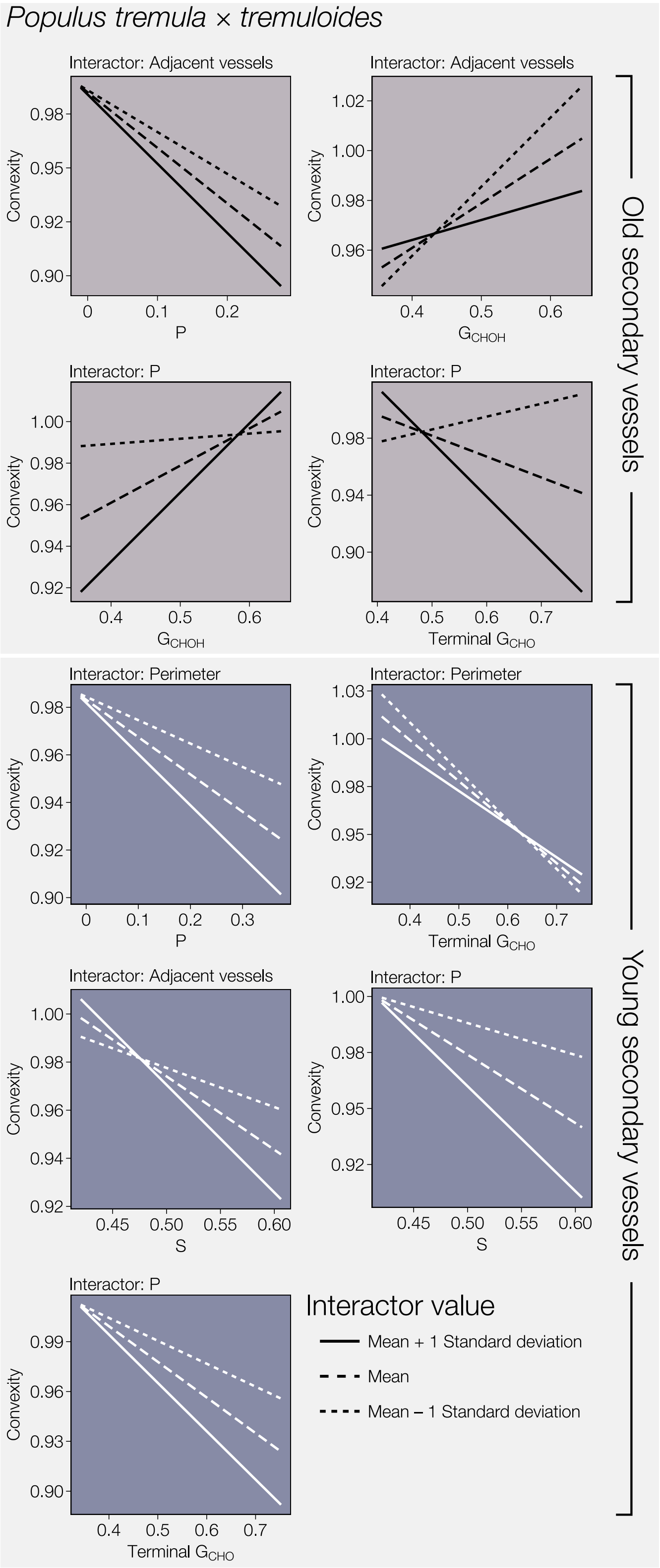

Supplemental figure S7 | Effects of cell wall morphology and composition on convexity in poplar are interdependent (related to figure 5).

Several two-way interactions between biochemical and morphological predictors significantly affected TE convexity. The lines represent effects of the respective variable on convexity when the interactor value was high (solid line), average (dashed line), or low (dotted line).

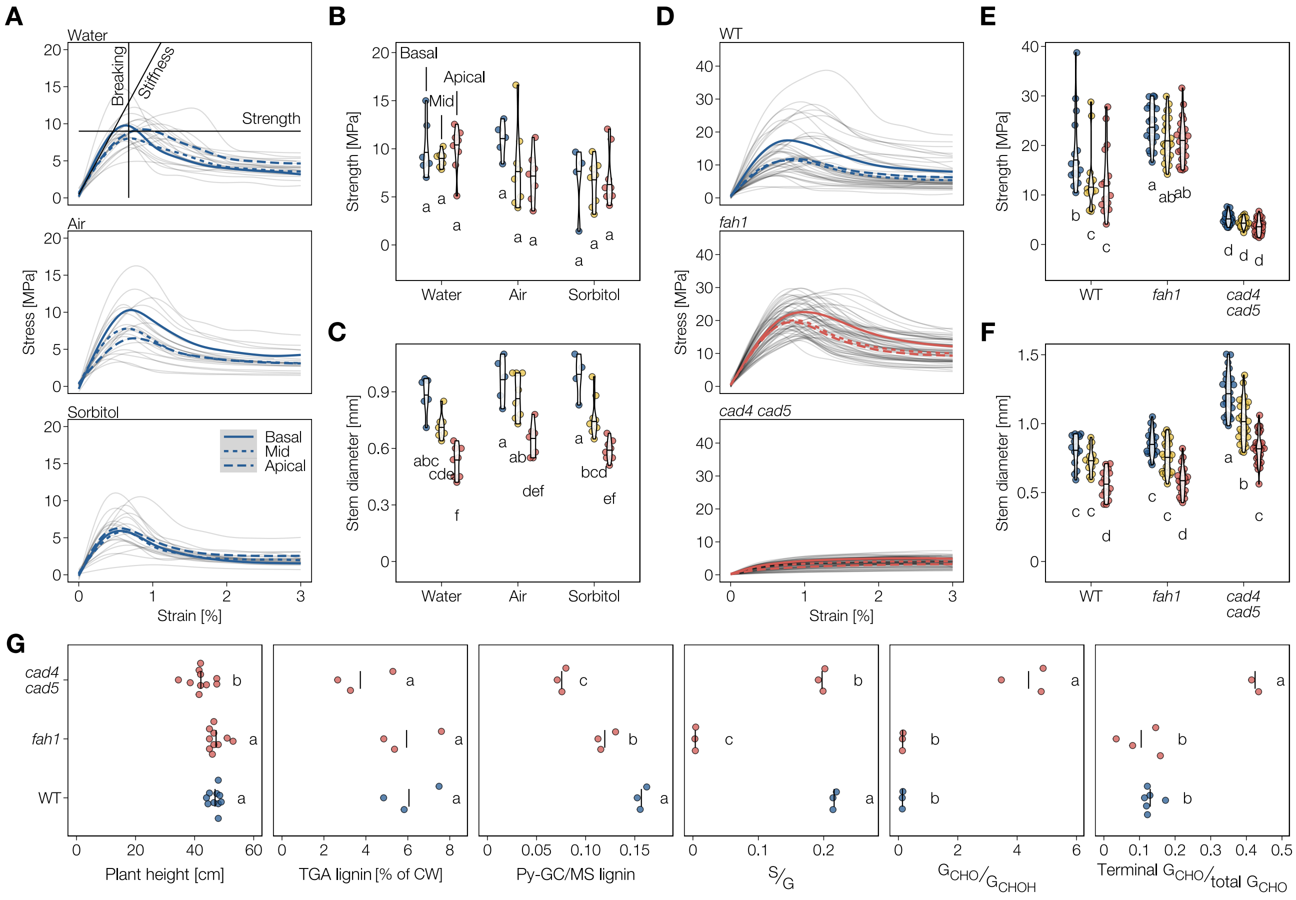

Supplemental figure S8 | Stem biomechanics and lignin composition in *fah1* and *cad4 cad5* (related to figure 6).
A Bending curves of the three-point-bending test of WT plants incubated in different solutions. Average bending curves for each developmental stage (basal, mid, apical) are indicated by blue lines. The strength is defined as the maximum endured stress (solid line), whereas stiffness is calculated from the linear phase of the elastic bending (dashed line). The point of breaking is defined as the amount of strain the stem can endure before catastrophic failure, and is visible as the peak of the bending curve (dotted line). **B** Flexural strength of WT stems tested after incubation in different media. **C** Diameters of WT stems used in the bending experiments assessing the influence of different media. **D** Bending curves of mutant plant stems. **E** Flexural strength of mutant plant stems. **F** Diameters of tested mutant plant stems. **G** Plant height, total lignin determined using thioglycolic acid derivatisation (TGA) and pyrolysis-GC/MS (Py-GC/MS), S/G and G_{CHO}/G_{CHOH} ratios determined using Py-GC/MS, and terminal G_{CHO}/G_{CHOH} ratio determined using thioacidolysis-GC/MS. Dots represent individual samples (single plants or pools of several plants), vertical lines represent the average for each variable and genotype. Different lowercase letters indicate significant differences according to a Tukey-HSD test (per panel; $\alpha = 0.05$).

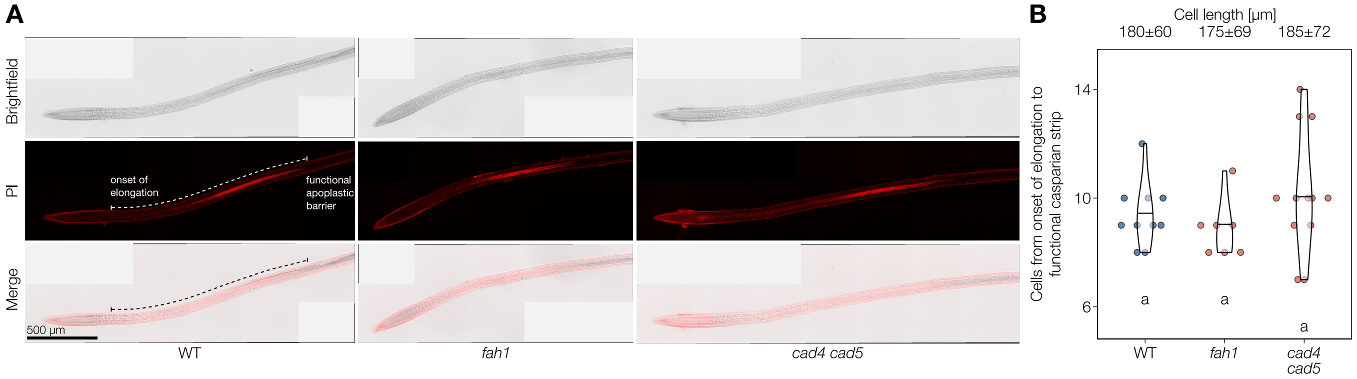

Supplemental figure S9 | Apoplastic barrier of the endodermis is unaffected in *fah1* and *cad4 cad5* (related to figure 7).
A Root tips of 4-day-old seedlings stained with propidium iodide (PI), indicating a functional apoplastic barrier in the endodermis excluding the dye from staining the vascular cylinder. **B** Establishment of the apoplastic barrier in n = 7–12 individual seedlings occurred roughly 10 cells after the onset of elongation, unaffected by mutations in *F5H* (*fah1*) or *CAD4* and *CAD5* (*cad4 cad5*) compared to WT plants. Different lowercase letters indicate significant differences according to a Tukey-HSD test (α = 0.05).

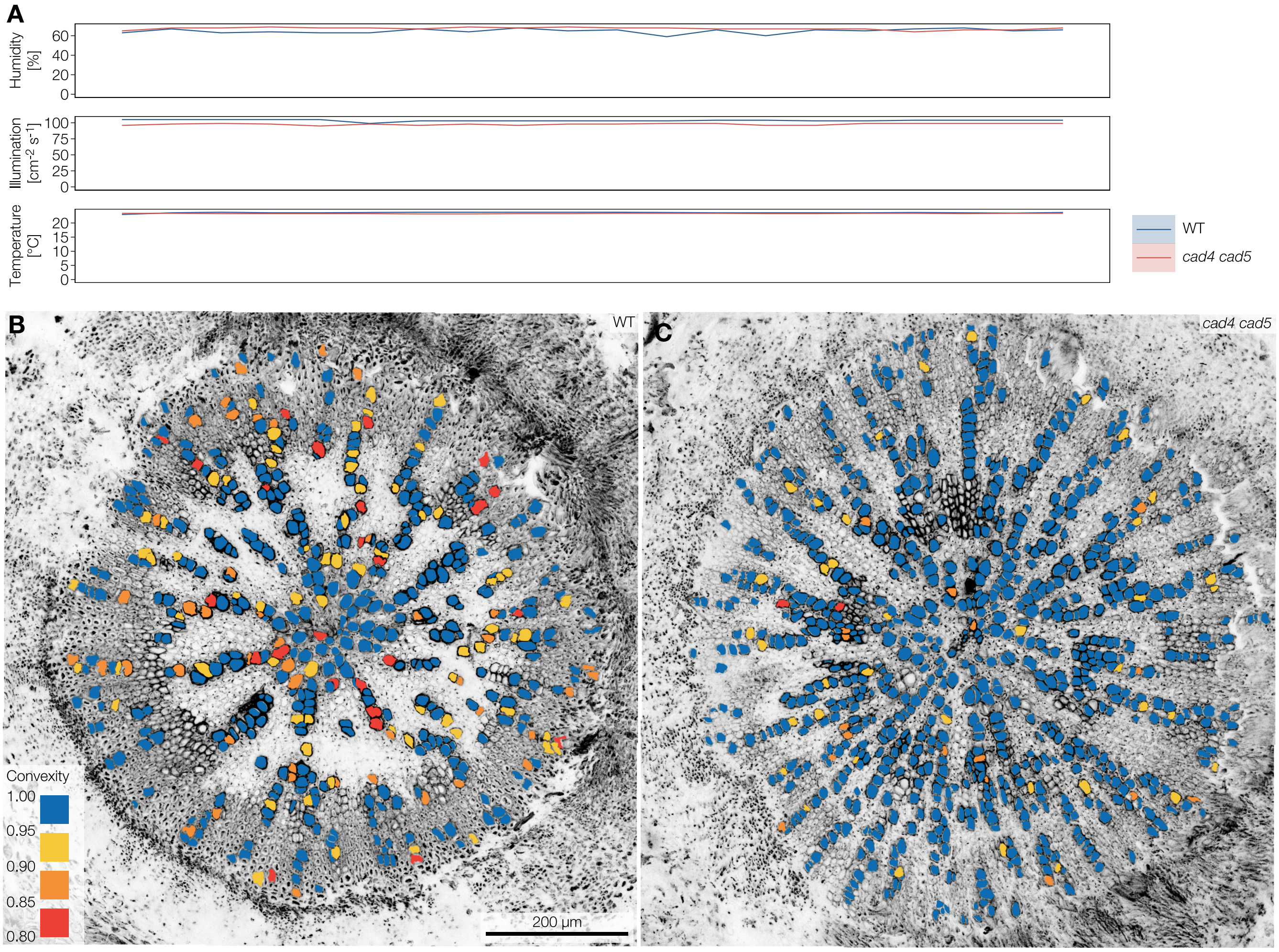

Supplemental figure S10 | Conditions and hypocotyl TE collapse during the drought experiment (related to figure 7).
A Environmental conditions during one of three independent instances of the simulated drought experiment. **B, C** Lignin autofluorescence micrographs of representative hypocotyl sections from the WT (**B**) and *cad4 cad5* (**C**) after simulated drought in 20% PEG followed by recovery in water. TEs are individually color-coded according to their convexity.

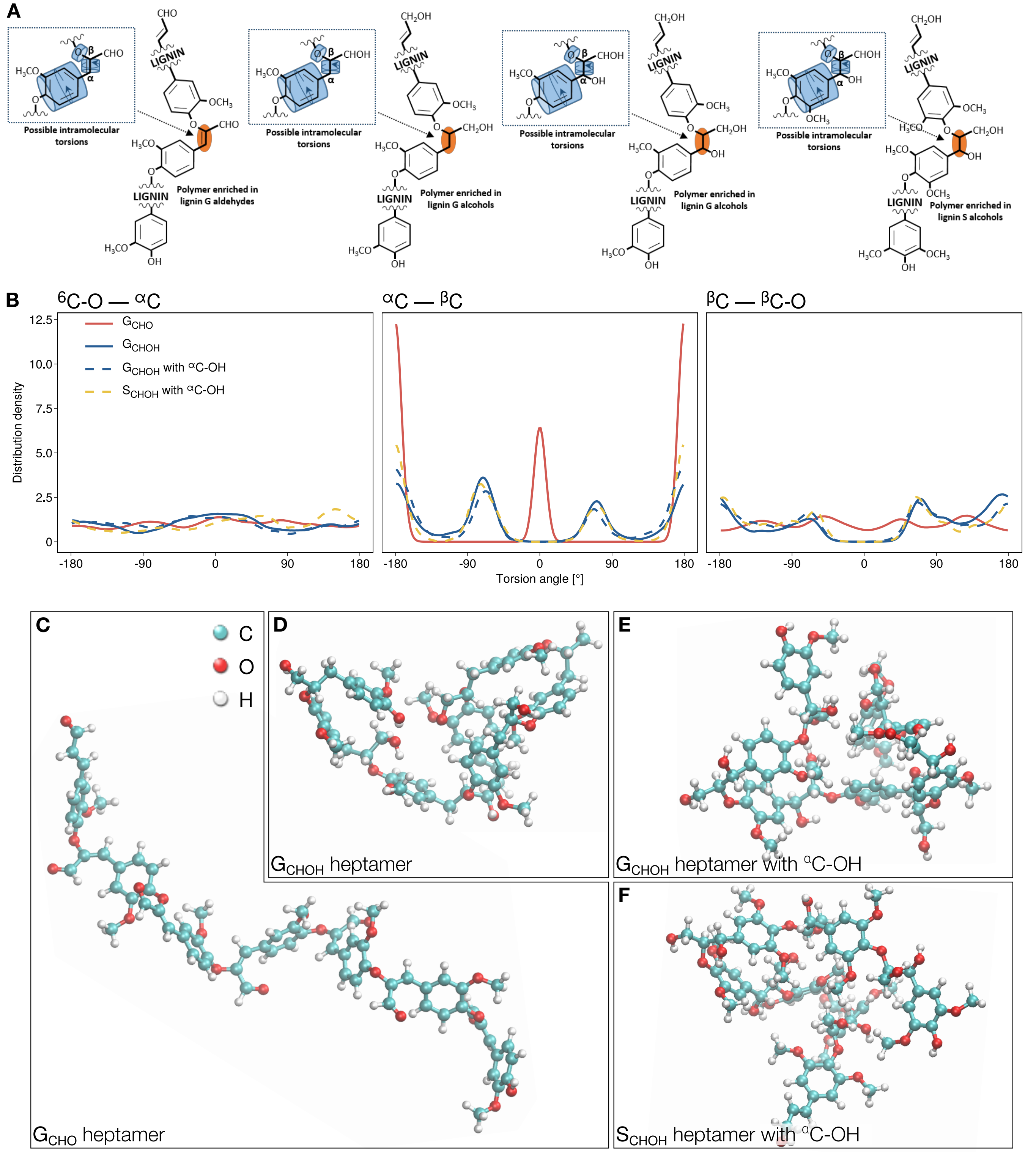

Supplemental figure S11 | Topology and mechanics of lignin oligomers depends on the C3 functional group (related to Table 1). **A** Schematic representation of the structure and torsion of ether linkage between residue of lignin oligomers depending on their aliphatic terminal function. **B** Distribution of torsion angles around the ^{6}C-O—$^\alpha$C, the $^\alpha$C—$^\beta$C and the $^\beta$C—$^\beta$C-O atoms for lignin oligomers made of G_{CHO} residues (red line), G_{CHOH} residues without (solid blue line) or with $^\alpha$C-OH (dotted blue line), or S_{CHOH} residues with $^\alpha$C-OH (dashed yellow line) determined from molecular dynamics simulations. **C–F** Typical molecular conformations from equilibrated molecular dynamics simulations of lignin heptamers with **G**$_{CHO}$ residues (**C**), G_{CHOH} residues without $^\alpha$C-OH (**D**), G_{CHOH} residues with $^\alpha$C-OH (**E**) and S_{CHOH} residues with $^\alpha$C-OH (**F**).

Supplemental table S1 | Used nomenclature of lignin chemistry.

C_6 ring substitution	Aliphatic length	Aliphatic function	Abbreviation	Name
G	C_3	–CHO	G_{CHO}	coniferaldehyde
G	C_3	–CHOH	G_{CHOH}	coniferyl alcohol
G	C_1	–CHO	benzaldehydes	vanillin
G	C_3	–CHO/–CHOH/–COOH	G	guaiacyl residues
S	C_3	–CHO/–CHOH/–COOH	S	syringyl residue
S	C_1	–CHO	benzaldehydes	syringaldehyde
P	—	—	P	phenyl residues

Supplemental table S2 | Insertional mutants and the targeted genes used in the present study, with gene name, locus number, number of paralog for each plant species and previous references in which these plants were analyzed.

Acronym	Locus	Full name	Plant species	Paralogs	Mutant alleles	Mutant ID	References
C4H	Potri.013G157900	*cinnamate-4-hydroxylase*	hybrid poplar	4			Bjurhager et al. 2010
4CL1	At1g51680	*4-coumarate-CoA ligase 1*	Arabidopsis	4	*4cl1-1*	SALK_142526	Van Acker et al. 2013
4CL2	At3g21240	*4-coumarate-CoA ligase 2*	Arabidopsis	4	*4cl2-4*	SALK_110197	Li et al. 2015
			Arabidopsis	4	*4cl1-1 4cl2-4*		Blaschek et al. 2020
CCoAOMT1	At4g34050	*caffeoyl-CoA O-methyltransferase 1*	Arabidopsis	1	*ccoaomt1*	SALK_151507	Kai et al. 2008
F5H1	At4g36220	*ferulate-5-hydroxylase 1*	Arabidopsis	2	*fah1-2*	EMS mutant	Meyer et al. 1998
OMT1	At5g54160	*caffeic acid O-methyltransferase 1*	Arabidopsis	1	*omt1*	SALK_135290	Tohge et al. 2007
CCR1	At1g15950	*cinnamoyl-CoA reductase*	Arabidopsis	2	*ccr1-3*	SALK_123-689	Mir Derikvand et al. 2008
CCR	Potri.003G181400	*cinnamoyl-CoA reductase*	hybrid poplar	15			Escamez et al. 2017
CAD4	At4g37980	*cinnamyl alcohol dehydrogenase 4*	Arabidopsis	9	*cad4-1*	SAIL_1265_A06	Lee et al. 2017
CAD5	At4g37990	*cinnamyl alcohol dehydrogenase 5*	Arabidopsis	9	*cad5-1*	SAIL_776_B06	Lee et al. 2017
			Arabidopsis	9	*cad4-1 cad5-1*		Blaschek et al. 2020

Paralog numbers were taken from Raes et al. (2003) for *Arabidopsis thaliana* and from Sundell et al. (2017) for hybrid poplar

Supplemental table S3 | Test statistics on global goodness-of-fit (Fisher's C) and directed separation (*i.e.* independence of variables) in the piecewise structural equation models (related to Figures 4 and 5).

Species	Cell type	Fisher's C	*P*-value[a]	Independence claim	Crit. value	*P*-value[b]
Arabidopsis	PX	16.650	0.034	Circularity ~ Perimeter + ...	-2.4405	0.0163
				Circularity ~ G_{CHOH} + ...	1.6967	0.0926
				Circularity ~ Terminal G_{CHO} + ...	-0.2495	0.8034
				Circularity ~ Total lignin + ...	1.2890	0.2001
	MX	23.020	0.003	Circularity ~ Total G_{CHO} + ...	1.0932	0.2769
				Circularity ~ S + ...	-2.4661	0.0153
				Circularity ~ Terminal G_{CHO} + ...	1.7678	0.0801
				Circularity ~ Total lignin + ...	2.2066	0.0296
	SX	6.996	0.136	Circularity ~ G_{CHOH} + ...	0.2350	0.8148
				Circularity ~ Benzaldehydes + ...	2.1184	0.0371
Poplar	Old SV	4.942	0.764	Circularity ~ P + ...	0.9855	0.3259
				Circularity ~ G_{CHOH} + ...	0.1769	0.8598
				Circularity ~ Terminal G_{CHO} + ...	0.8703	0.3855
				Circularity ~ Total lignin + ...	0.2767	0.7824
	Young SV	13.135	0.216	Circularity ~ Perimeter + ...	2.2784	0.0240
				Circularity ~ P + ...	0.8344	0.4052
				Circularity ~ G_{CHOH} + ...	-0.2598	0.7954
				Circularity ~ S + ...	-1.2235	0.2229
				Circularity ~ Terminal G_{CHO} + ...	-0.2332	0.8159

PX, protoxylem TEs; MX, metaxylem TEs; SX secondary xylem TEs; PV, primary vessels; SV, secondary vessels
Independence claims are formatted as (response variable) ~ (predictor variable) + ... (*i.e.* within the models defined in Figs. 4 and 5)

[a] From the global goodness-of-fit test; values < 0.05 indicate that a theoretically better model can be built by including additional relationships between parameters.

[b] From the test of directed separation; values < 0.05 indicate that the respective relationship is statistically significant.

Supplemental table S4 | Standardised and raw coefficients and their *P*-values in the piecewise structural equation models (related to figures 4 and 5).

Species	Cell type	Response	Predictor	Estimate	Std. error	Crit. value	*P*-value	Std. estimate[a]
Arabidopsis	PX	Circularity	Convexity	1.9018	0.0633	30.0486	0.0000	0.9441
		Convexity	Perimeter	0.0078	0.0012	6.5366	0.0000	0.4690
		Convexity	G_{CHOH}	0.1949	0.0536	3.6377	0.0004	0.2674
		Convexity	Terminal G_{CHO}	0.1228	0.0435	2.8237	0.0057	0.2041
		Convexity	Total lignin	19.5650	6.8561	2.8537	0.0052	0.2045
	MX	Circularity	Adjacent vessels	-0.0537	0.0274	-1.9615	0.0525	-0.0677
		Circularity	Convexity	2.0009	0.0748	26.7501	0.0000	0.9231
		Convexity	Adjacent vessels	-0.0626	0.0270	-2.3181	0.0225	-0.1712
		Convexity	Total G_{CHO}	0.2336	0.0891	2.6216	0.0101	0.2172
		Convexity	S	-0.3491	0.1299	-2.6872	0.0084	-0.2950
		Convexity	Terminal G_{CHO}	0.1427	0.0610	2.3416	0.0212	0.1902
		Convexity	Total lignin	17.0394	8.1287	2.0962	0.0386	0.2237
	SX	Circularity	Convexity	2.0475	0.1507	13.5842	0.0000	0.8290
		Convexity	G_{CHOH}	0.0660	0.0264	2.5042	0.0142	0.2221
		Convexity	Benzaldehydes	-0.1178	0.0195	-6.0314	0.0000	-0.5349
Poplar	PV	Circularity	Adjacent vessels	-0.0111	0.0208	-0.5327	0.5955	-0.0306
		Circularity	Convexity	3.6455	0.2520	14.4680	0.0000	0.8312
		Convexity	Adjacent vessels	-0.0164	0.0083	-1.9707	0.0517	-0.1982
	Old SV	Circularity	Adjacent vessels	-0.0741	0.0141	-5.2637	0.0000	-0.2209
		Circularity	Convexity	2.0701	0.1097	18.8682	0.0000	0.7918
		Convexity	Adjacent vessels	-0.0218	0.0079	-2.7535	0.0066	-0.1696
		Convexity	P	-0.1817	0.0247	-7.3561	0.0000	-0.5321
		Convexity	G_{CHOH}	0.1282	0.0505	2.5380	0.0121	0.3109
		Convexity	Terminal G_{CHO}	-0.0795	0.0360	-2.2090	0.0287	-0.2481
		Convexity	Total lignin	6.6005	2.4852	2.6559	0.0087	0.2019
	Young SV	Circularity	Adjacent vessels	-0.1153	0.0131	-8.8058	0.0000	-0.3740
		Circularity	Convexity	1.9740	0.1256	15.7185	0.0000	0.6675
		Convexity	Adjacent vessels	-0.0180	0.0064	-2.8013	0.0057	-0.1731
		Convexity	Perim.	-0.0002	0.0001	-2.3985	0.0176	-0.1620
		Convexity	P	-0.1195	0.0161	-7.4287	0.0000	-0.4652
		Convexity	G_{CHOH}	0.2119	0.0404	5.2477	0.0000	0.6687
		Convexity	S	-0.3164	0.0490	-6.4540	0.0000	-0.4228
		Convexity	Terminal G_{CHO}	-0.2424	0.0374	-6.4732	0.0000	-0.8465

PX, protoxylem TEs; MX, metaxylem TEs; SX secondary xylem TEs; PV, primary vessels; SV, secondary vessels

[a] Standardised coefficients allow direct comparisons of relative impacts within each model, but not across different models.

Supplementary movie S1 | Movie showing the three-point bending set-up used to measure the biomechanical properties of Arabidopsis stem segments.

Bibliography

Bjurhager, I., A.-M. Olsson, B. Zhang, L. Gerber, M. Kumar, L. A. Berglund, I. Burgert, B. Sundberg, and L. Salmén. 2010. Ultrastructure and Mechanical Properties of *Populus* Wood with Reduced Lignin Content Caused by Transgenic Down-Regulation of Cinnamate 4-Hydroxylase. *Biomacromolecules* 11, no. 9 (2010): 2359–2365. https://doi.org/10.1021/bm100487e.

Blaschek, L., A. Champagne, C. Dimotakis, Nuoendagula, R. Decou, S. Hishiyama, S. Kratzer, S. Kajita, and E. Pesquet. 2020. Cellular and Genetic Regulation of Coniferaldehyde Incorporation in Lignin of Herbaceous and Woody Plants by Quantitative Wiesner Staining. *Frontiers in Plant Science* 11:109. https://doi.org/10.3389/fpls.2020.00109.

Escamez, S., M. L. Gandla, M. Derba-Maceluch, S.-O. Lundqvist, E. J. Mellerowicz, L. J. Jönsson, and H. Tuominen. 2017. A collection of genetically engineered *Populus* trees reveals wood biomass traits that predict glucose yield from enzymatic hydrolysis. *Scientific Reports* 7, no. 1 (2017): 1–11. https://doi.org/10.1038/s41598-017-16013-0.

Kai, K., M. Mizutani, N. Kawamura, R. Yamamoto, M. Tamai, H. Yamaguchi, K. Sakata, and B. I. Shimizu. 2008. Scopoletin is biosynthesized via *ortho*-hydroxylation of feruloyl CoA by a 2-oxoglutarate-dependent dioxygenase in *Arabidopsis thaliana*. *Plant Journal* 55 (6): 989–999. https://doi.org/10.1111/j.1365-313X.2008.03568.x.

Lee, S., H. Mo, J. I. Kim, and C. Chapple. 2017. Genetic engineering of *Arabidopsis* to overproduce disinapoyl esters, potential lignin modification molecules. *Biotechnology for Biofuels* 10 (1): 40. https://doi.org/10.1186/s13068-017-0725-0.

Li, Y., J. I. Kim, L. Pysh, and C. Chapple. 2015. Four Isoforms of Arabidopsis 4-Coumarate:CoA Ligase Have Overlapping yet Distinct Roles in Phenylpropanoid Metabolism. *Plant Physiology* 169 (4): 2409–2421. https://doi.org/10.1104/pp.15.00838.

Meyer, K., A. M. Shirley, J. C. Cusumano, D. A. Bell-Lelong, and C. Chapple. 1998. Lignin monomer composition is determined by the expression of a cytochrome P450-dependent monooxygenase in *Arabidopsis*. *Proceedings of the National Academy of Sciences* 95 (12): 6619–6623. https://doi.org/10.1073/pnas.95.12.6619.

Mir Derikvand, M., J. B. Sierra, K. Ruel, B. Pollet, C.-T. Do, J. Thévenin, D. Buffard, L. Jouanin, and C. Lapierre. 2008. Redirection of the phenylpropanoid pathway to feruloyl malate in *Arabidopsis* mutants deficient for cinnamoyl-CoA reductase 1. *Planta* 227, no. 5 (2008): 943–956. https://doi.org/10.1007/s00425-007-0669-x.

Noda, L. K., and O. Sala. 2000. A resonance Raman investigation on the interaction of styrene and 4-methyl styrene oligomers on sulphated titanium oxide. *Spectrochimica Acta Part A: Molecular and Biomolecular Spectroscopy* 56, no. 1 (2000): 145–155. https://doi.org/10.1016/S1386-1425(99)00128-6.

Raes, J., A. Rohde, J. H. Christensen, Y. Van de Peer, and W. Boerjan. 2003. Genome-Wide Characterization of the Lignification Toolbox in Arabidopsis. *Plant Physiology* 133, no. 3 (2003): 1051–1071. https://doi.org/10.1104/pp.103.026484.

Sundell, D., N. R. Street, M. Kumar, E. J. Mellerowicz, M. Kucukoglu, C. Johnsson, V. Kumar, C. Mannapperuma, N. Delhomme, O. Nilsson, H. Tuominen, E. Pesquet, U. Fischer, T. Niittylä, B. Sundberg, and T. R. Hvidsten. 2017. AspWood: High-Spatial-Resolution Transcriptome Profiles Reveal Uncharacterized Modularity of Wood Formation in *Populus tremula*. *The Plant Cell* 29 (7): 1585–1604. https://doi.org/10.1105/tpc.17.00153.

Tohge, T., K. Yonekura-Sakakibara, R. Niida, A. Watanabe-Takahashi, and K. Saito. 2007. Phytochemical genomics in *Arabidopsis thaliana*: A case study for functional identification of flavonoid biosynthesis genes. *Pure and Applied Chemistry* 79 (4): 811–823. https://doi.org/10.1351/pac200779040811.

Van Acker, R., R. Vanholme, V. Storme, J. C. Mortimer, P. Dupree, and W. Boerjan. 2013. Lignin biosynthesis perturbations affect secondary cell wall composition and saccharification yield in *Arabidopsis thaliana*. *Biotechnology for Biofuels* 6 (1): 46. https://doi.org/10.1186/1754-6834-6-46.
