# Supplementary material for: Plant biomechanics and resilience to environmental changes are controlled by specific lignin chemistries in each vascular cell type and morphotype
Source: Plant Cell. 2022 Sep 21;34(12):4877–96. doi: 10.1093/plcell/koac284 (PMC9709985; doi:10.1093/plcell/koac284)
Supplement: koac284_Supplementary_Data [file tpc21.00813.PeerReviewReport.pdf › tpc21.00813.PeerReviewReport.pdf]

Plant biomechanics and resilience to environmental changes are controlled by specific lignin chemistries in each vascular cell type and morphotype

Delphine Ménard, Leonard Blaschek, Konstantin Kriechbaum, Cheng Choo Lee, Henrik Serk, Chuantao Zhu, Alexander Lyubartsev, Nuoendagula, Zoltán Bacsik, Lennart Bergström, Aji Mathew, Shinya Kajita, and Edouard Pesquet

Corresponding author: Edouard Pesquet (edouard.pesquet@su.se).

Review timeline:

**TPC2021-RA-00648**	Submission received:<br>1st Decision:	July 28, 2021<br>September 19, 2021 *manuscript declined*
**TPC2021-RA-00813**	Submission received:<br>1st Decision:	May 4, 2022<br>June 5, 2022 *revision requested*
**TPC2021-RA-00813R1**	1st Revision received:<br>2nd Decision:<br>Final acceptance:	July 23, 2022<br>August 24, 2022 *acceptance pending, sent to sci editor*<br>Sept. 11, 2022

REPORT: (The report shows the major requests for revision and author responses. Minor comments for revision and miscellaneous correspondence are not included. The original format may not be reflected in this compilation, but the reviewer comments and author responses are not edited, except to correct minor typographical or spelling errors that could be a source of ambiguity.)

TPC2021-RA-00537 1st Editorial decision – *declined* **September 19, 2021**

Thank you for choosing to send your manuscript entitled "Specific and dynamic lignification at the cell-type level controls plant physiology and adaptability" for consideration at The Plant Cell. Your submission has been evaluated by members of the editorial board as well as expert reviewers in your field, and we regret to inform you that we are not able to recommend publication of this manuscript in its current form. We have not made this decision lightly. We have had input from multiple scientists, and we have solicited post-review comments as well. Our present policy is to offer streamlined decisions and to not advise on the direction of the work by requesting extensive modifications or substantial additional experiments.

During the post-review consultation session, we also agreed that if you could address the major points raised by the reviewers by new experiments, we would welcome a resubmission. This may be treated as a new submission, but we would attempt to use at least some of the same reviewers. Nevertheless, reviewers will be asked to assess as a new manuscript (i.e. are the claims fully supported by the data and do the results presented move the field forward?), and not only whether previous reviewer comments have been addressed.
The reviewers have raised many important points. A general theme of these points is that various results have been over-interpreted and alternative explanations have not been sufficiently considered. Addressing these will require both more experiments and thorough rewriting of the manuscript. In addition, the reviewers and editors request that you remove all claims of priority (first to do . . .). Please also reference and discuss the preceding literature on how lignification affects xylem function in plants; in fact, a lot is known.

It will be important to convince the editors and reviewers that the study adds significant new understanding(s) of mechanisms or processes, or otherwise represents a significant advance in the field, and that the major claims made are fully justified by the data presented. This includes careful consideration and explanation of the various controls used in experiments, the extent and manner of replication, and the statistical analyses used. Sampling methods and the nature of "biological replicates" should be described precisely (i.e. different plants, parts of plants, pooled tissue, independent pools of tissue, sampled at different times, etc.), along with a clear description of and rationale for any statistical analyses conducted. The reader should know exactly what was sampled; what forms the basis of the calculation of any means and other statistical variables and parameters reported. This is also necessary to ensure that proper statistical analysis was conducted.

-- Reviewer comments:

TPC2021-RA-00813 Submission received **May 4, 2022**

Reviewer comments on previously declined manuscript and **author responses:**

Reviewer #1:

This manuscript used original and state of the art methods for investigating the relationship between lignin composition and biomechanical properties. The manuscript is well organised and have high quality figures. This work represents a clear advance in the field of lignin and open new avenues to investigate the structure and function of lignin.

First the authors used atomic force microscopy and different drying methods on plant pluripotent cell suspension cultures. They showed that lignin deposition of TE is directly associated with biomechanical resistance.
Then, the authors showed that the different lignified cell types of Arabidopsis stem have a specific lignin concentration and composition. Changes in lignin composition observed in phenylpropanoid pathway mutants is linked with morphological changes of the different TEs. The authors used computational modelling to determine to determine the role of the different lignin residues on the biomechanical properties of each TE morphotype. This reveals that the different lignin residues differentially affect the morphological properties of the different TE morphotypes.

The authors performed similar analysis using the stem of a woody plant, poplar and showed again that lignin structure have specific effect on the morphological/biomechanical properties of the different TE morphotypes.
By using Arabidopsis mutants affected by changes in lignin composition and concentration (fah1 and cad4x5 mutants), the authors showed that S/G ratio controls the stem stiffness and whereas GCHO/GCHOH controlled stem flexibility.

Finally, the authors showed that the mutants cad4x5 mutant have an increased resistance to, and recovery from, simulated drought.

Point 1. The results presented in Fig. 6 nicely show the consequence of lignin modification on mechanical properties. In lines 252-253, the authors state that GCHO/GCHOH controlled stem flexibility. They showed that cad4x5 mutant exhibit an increased GCHO/GCHOH and an increased flexibility. The cad4x5 mutant also display a reduction of ~40% of total lignin compared to WT. Could the increased stem flexibility be due to the decreased lignin content and not to the increased GCHO/GCHOH?

RESPONSE: Excellent comment from the reviewer as it is practically impossible to modify lignin composition in whole plants without affecting its amounts. This difficulty is due to both biological compensation (as we shown in Blaschek et al., 2020) but also with current quantification methods which cannot precisely quantify lignin which are approximated (see Fig S7 where we provided two different methods to quantify lignin; this quantification difficulty has been discussed by Hatfield and Fukushima, 2005 https://doi.org/10.2135/cropsci2004.0238). We have however solved the issues by performing molecular dynamic simulations of lignin heptamers made of either GCHO or GCHOH. Using the same amount of heptamers in a defined volume, we confirmed that it is not the amount but the nature of the terminal aliphatic functions which alter the biomechanics of lignin. This was added in a new paragraph entitled "Increasing coniferaldehyde residues in lignin changes molecular conformations, torsions and reduces stiffness compared to coniferyl alcohol residues" supported by Table 1 and Fig. S10.

Point 2. In the Fig. 7 and in the last paragraph of the result section (Line 255-271), the authors claimed that TE flexibility due to lignin composition enables plants to better resist drought. This claim is supported by the cad4x5 mutant displaying both an increased flexibility of stem (Fig. 6F) and an increased resistance to drought (Fig7) compared to WT. I am not sure if the increased flexibility observed in stem can explain the increased resistance to drought observed in leaves. The mutation in CAD4&5 can modify the water transport capacity and plant physiology independently of the TEs lignification. The root endodermal cells are also lignified and are critical in controlling water transport. It has been shown that a similar mutant (ccr1/cad4&5) displays a defective Casparian strip (1). Casparian strip defect is known to trigger several compensatory mechanisms such as increased lignin/suberin deposition in the endodermis, stomata closures and aquaporin inactivation as observed in other Casparian strip mutants (2-4). This is accompanied by an improved water used efficiency and drought tolerance (5). Can the authors discard that the increased drought resistance observed in cad4&5 is not due to the consequence of a putative defective Casparian strip? This could be done by checking the functionality of the Casparian strip in cad4x5 mutant using an apoplastic tracer, propidium iodide. (6).

RESPONSE: We thank the reviewer for this comments and have accordingly checked for any defect in apoplastic barrier in the *fah1* and *cad4xcad5* mutants compared to the WT plants using the suggested propidium iodine test but did not observe any difference. This result showed that the increased drought tolerance observed for *cad4xcad5* is not due to Casparian strip defects. This was added to the paragraph entitled "Increased TE cell wall flexibility due to coniferaldehyde in lignin enables plants to better recover from drought" and supported by Fig. S8.

Point 3. Title: I am not sure if the term "plant physiology" should be kept in the title. This manuscript is mainly focus on cellular features rather than plant physiology.

RESPONSE: We have accordingly changed the title to "Plant biomechanics and resilience to environmental changes are controlled by specific lignin chemistries in each vascular cell type and morphotype".

Point 4. The figure 1C compares the EDS ratio of PCW and SCW. There is no mention of the EDS ratio of PCW in the text (line 111-124). The text only mentions the increased lignification over the time in the SCW. Why are the EDS ratio of PCW are showed in the figure? Would it be more relevant to show a statistical analysis comparing the different time points (Days after induction)?

RESPONSE: We thank the reviewer for this comment and have accordingly performed statistical analysis between both time, cell wall layer as well as morphotype. This was added to the paragraph entitled "Post-mortem accumulation of lignin increases the cell wall stiffness of isolated TEs morphotypes" and supported by Fig. 1C and S2C.

Point 5. Figure 3B and line 151-154. Could the author better explain why they measured the relative proportion of adjacent cell type for each TE morphotype? Why is this measurement relevant for this manuscript? (Same question for Fig. 5B.)

RESPONSE: As sap conduction in xylem occurs through dead TE lumens, the entire vascular tissue is associated to plant hydraulics with XP, and potentially also XF, providing essential osmotic pressure to laterally distribute the sap content. TE grouping has indeed been previously suggested to play an important role in xylem hydraulics (see Martínez-Vilalta et al., 2012) and we therefore investigated the contribution of surrounding cell types to each TE morphotypes. This was added to the introduction and the discussion.

Point 6. Could the author explicit their nomenclature of the lignin residues (ex: C6 and C3). Different nomenclatures exist in the literature, this can be confusing for non-lignin expert. This could be added to Fig S1.

RESPONSE: Lignin chemistry is generally limited to its aromatic residues (i.e. H, G, 5H, S,…) independently of their position in the polymer or their aliphatic functions, we thus used a more precise nomenclature used for phenylpropanoid (from the IUPAC recommendation; Moss, 2000) to include both aromatic and aliphatic part of the canonical phenylpropanoid residues as previously used for lignin by Lapierre et al., (1993). To ease the reading, we thus added Table S1 with the various acronyms corresponding to each residue.

Point 7. The authors measured vessel morphology and cell wall composition (Fig. 4, S2 and S3). The authors state that "specific changes in lignin, altering the C6 and C3 parts of lignin residues, differently control the biomechanical properties of each TE morphotype" (Line 177-178). Could the authors better explain the link between vessel morphology and biomechanical property?

RESPONSE: As xylem sap is rising due to a negative pressure, optimal hydraulics is observed for circular conduits and thus impaired if the TE inwardly collapses. The maintenance of optimal lumen morphology in dead sap conducting TEs thus depends on the biomechanics of its cell walls. We confirmed the link between TE morphology/cell wall composition and its biomechanics using iPSCs with different drying methods as well as with AFM in Figure 1 and 2. Clarifications were added in paragraphs entitled "Post-mortem accumulation of lignin increases the cell wall stiffness of isolated TEs morphotypes" and "Post-mortem lignin accumulation controls the resistance of isolated TE morphotypes to negative pressure" as well as in the introduction.

Point 8. Why does the authors used cell circularity as a readout of biomechanical properties?

RESPONSE: We used both circularity and convexity (inward collapse) as the normal readout of TE deformation due to a negative pressure – similar inward collapse were previously shown to occur under extreme drought (see Cochard et al., 2004; Zhang et al., 2016).

Point 9. Lines 181-182: "Only S residue content escalated collapse, whereas GCHO position and the proportion of neighbouring TEs had no effect (Fig. 4B)." According to Fig. 4B, S residue content does not change the convexity and circularity in the PX.

RESPONSE: We agree and have revised the sentence accordingly.

Point 10. Why H residues were not measured in this manuscript?

RESPONSE: There are currently no *in situ* imaging method capable of reliably and directly quantifying H residue in lignin. We have already checked and published this for Raman spectroscopy in Blaschek et al., (2020). This limitation was added in the discussion.

Point 11. Could the authors explain what the P residues are?

RESPONSE: We can detect unsubstitued aromatic residues (call phenyl) using Raman spectroscopy (see Noda and Sala, 1999 and Fig S3A) which derive from lignin during polymerisation (Kawamoto, 2017) and/or from side groups of non-canonical residue such as stilbenoids, flavonoids or coumaroyl esters.

<u>Reviewer #2:</u>

Menard et al. present a set of result on tracheary element, making links between lignin composition, shape, stiffness, and hydraulic conductivity. All the hypotheses presented are highly interesting and may renew our view of the function of lignification.

Point 1. However, the paper is very broad in terms of concepts and functions, while 3 systems are studied (Arabidopsis cell culture, Arabidopsis stems, poplar stems); this makes it difficult to reach strong conclusions.

RESPONSE: We consider that the uses of three different systems, all complementary and used to understand the same cell type, provided independent lines of evidence converging to strengthen the conclusion of our manuscript. One system alone cannot answer the various aspect of lignin in specific TE morphotypes at distinct stage of maturation as single cells and within a tissue context. We thus fully investigated TE at the single cell level using three complementary system including (i) inducible cell culture to observe isolated TEs at different stage of maturation and alter their biochemistry and external conditions, (ii) TEs in *Arabidopsis* vascular bundles to investigate morphotypes in tissues and alter their biochemistry and external conditions, and (iii) TEs to integrate TE morphotypes in tissues during different stages of maturation and alter their biochemistry.

Point 2. The paper is overall clearly written and organised, though the overuse of acronymes makes it sometimes difficult to follow the flow.

RESPONSE: We understand and have added table S1 and S2 to ease the understanding of the various acronyms corresponding to mutated/modulated genes as well as the various residues of lignin.

Point 3. Lines 123-124, the authors unduly conclude that they show that lignification continues 40 days after cell death. They only show changes in C/O ratios, which is not specific of lignin. Additional assessment of lignin content is needed.

RESPONSE: We agree with the reviewer and have accordingly measured lignin during the TE *post-mortem* maturation using the thioglycolic acid derivatisation method and confirmed the reliability of this method for lignin with samples treated with piperonylic acid, which showed no detectable lignin increase. This was added to the paragraph entitled "Post-mortem accumulation of lignin increases the cell wall stiffness of isolated TEs morphotypes" and presented in Fig. S2D.

Point 4. Lines 177-178. It is claimed that alterations of cell shape (irregular xylem phenotype) are due to alterations of lignin contents. This phenotype could occur before cell death. For instance, mutations in CESA7 induce irregular xylem, but do not alter lignin patterns (Takenaka et al. Plant Cell 2018). The authors need to analyse cell shape at earlier developmental stages or following draught.

RESPONSE: We thank the reviewer and have clarified that the inward collapse of TEs can only be observed after death in a sap conducting context, so no need to study cell shape prior to TE cell death. As we show that lignin is incorporated onto the polysaccharide network of the secondary cell walls, altering this network before cell death will consequently affect the deposition of lignin. We have thus added multiple references showing that the importance of death and sap conduction in both inducible cell culture and ectopic transdifferentiation (Endo et al., 2008; Takenaka et al., 2018) in the introduction. As requested by the reviewer, we also evaluated changes in TE shape following drought treatment and showed a significant increase in inward collapse with drought in WT plants. This was added to the paragraph entitled "Increased TE cell wall flexibility due to coniferaldehyde in lignin enables plants to better recover from drought" and presented in Fig. S9B-C.

Point 5. Lines 177-178. It is implicitly stated that changes in cell shape are equivalent to changes in biomechanical properties. This is certainly not true: fah1 has no alterations of cell shape but shows differences in stiffness.

RESPONSE: We thank the reviewer and have clarified this statement in the paragraph entitled "Lignin concentration and composition differently affects the resistance to negative pressure of each TE morphotype in annual plants".

Point 6. Work on the three systems is somehow disconnected. Why didn't the authors produce cell cultures from some of the extreme Arabidopsis mutants to test more directly the effect of lignin composition on cell wall mechanics and tracheary element collapse?

RESPONSE: We thank the reviewer and clarified why we used these three complementary systems. As TEs are dead conducting cells, their function relies on both cell autonomous properties as well as cooperative aspects in tissues, which vary with morphotypes and developmental stages. We thus fully investigated TE at the single cell level using three complementary system including (i) inducible cell culture to observe isolated TEs at different stage of maturation and alter their biochemistry and external conditions, (ii) TEs in Arabidopsis vascular bundles to investigate morphotypes in tissues and alter their biochemistry and external conditions, and (iii) TEs to integrate TE morphotypes in tissues during different stages of maturation and alter their biochemistry.

Point 7. The paragraphs lines 179-199 and 215-233 are written as if structural equation models demonstrated causal relations between variables. The authors only show associations between lignin composition/content and cell geometry and assess the strength of these associations.

RESPONSE: We thank the reviewer and clarified our statement in the paragraph entitled "Lignin concentration and

composition differently affects the resistance to negative pressure of each TE morphotype in annual plants". As our multiple experiments show that lignin directly affects the resistance/susceptibility to inward collapse, we used a large array of variation in morphology and cell wall biochemistry for each TE morphotypes to define the relationship between lignin chemistry and inward collapse using structural equation modelling. To confirm the potential causal relationships defined by the structural equation modelling, we performed molecular dynamic simulations to only test one parameter (alcohol vs aldehyde) and confirmed that this difference in lignin residues corresponded to what we observed.

Point 8. 3-point bending test. Results with the cad4x5 are difficult to interpret: what are the relative contributions of changes in the cell wall and changes in cell shape to the reduction in modulus?

RESPONSE: We agree with the reviewer and have clarified this aspect by making the three-point bending on three different portion of Arabidopsis stems which display difference in xylem development. Previous studies have shown that apical portion of the stem mostly include TEs in contrast to basal portion which include fibers (Hoffmann et al., 2020; Morel et al., 2022). Using this developmental gradient, we could compare the impact of turgor pressure and of lignin composition on the mechanical properties using three-point bending. We thus confirmed that the observed changes in stiffness or flexibility due to specific lignin changes mostly affected the apical portion, in line with our previous observation on TE biomechanics at the single cell level. This was added to the paragraph entitled "Aliphatic and aromatic changes in lignin residues non-redundantly control distinct mechanical properties of plant stems" and presented in Fig. 6 and S7.

Point 9. Lines 270-271. The authors claim that lignin structure controls hydraulic properties. This is too strong a conclusion based on a single mutant. It is feasible to implement the assay of Fig. 7D in several mutant lines.

RESPONSE: We agree with the reviewer and have clarified this aspect as we cannot define whether it is an improved resistance or an improved recovery. As any deformation or inward collapse of conduits will affect its hydraulic properties (see Hagen-Poiseuille law for fluid dynamics), we did not need to perform our analysis on a set of mutants but instead focused on the TE morphology of WT and mutant plants following drought. This was added to the paragraph entitled "Increased TE cell wall flexibility due to coniferaldehyde in lignin enables plants to better recover from drought" and presented in Fig. S9B-C.

Point 10. Please reduce the number of acronyms. For instance, iPSC may be replaced by cell culture.

RESPONSE: We agree with the reviewer and have supplied supplementary table S1 and S2 to help the reading of the different acronyms for lignin residues and gene names. As for the cell culture, we used the terminology of cell biology according to previous call to standardise the naming and reporting of cell cultures (Luong et al., 2011 https://doi.org/10.1016/j.stem.2011.03.002) where immortalised cell cultures (uncapable of differentiating) and inducible pluripotent cell cultures (capable of differentiating in different cell types depending on the trigger – Ménard et al., 2017 / Dubreuil et al., 2019) need to be clearly distinguish.

Point 11. I don't understand lines 70-71: the matric potential generally refers to the soil; what is mean with 'contribution of mechanical pressure exerted by TE'?

RESPONSE: Good comment as matric potential has different meaning depending if you are a soil chemist (referring to colloidal structure of soil) or plant physiologist (referring to cell wall spaces and their biomechanics). We removed any reference of the matric potential in the revised text to remove ambiguity.

Point 12. line 114 please specify the species from which cells were cultured

RESPONSE: We thank the reviewer and have accordingly revised the text to state that iPSCs derived from Arabidopsis thaliana Col-0 (same as our WT Arabidopsis). This was added in the paragraph entitled "Post-mortem accumulation of lignin increases the cell wall stiffness of isolated TEs morphotypes".

Point 13. In Fig 1D, the scales are missing for the quantities measured.

RESPONSE: Great comment, for Fig 1D we added the range corresponding to our color coding. Additionally, as there are no absolute quantification method for lignin (no extinction coefficient, molecular weight,… see Hatfield and Fukushima, 2005 https://doi.org/10.2135/cropsci2004.0238), we calibrated our *in situ* lignin quantification on known mutants previously shown to exhibit specific changes in lignin amount and/or composition.

Point 14. Atomic force microscopy. How did the authors differentiate secondary from primary cell wall? Please provide raw data separately for primary and secondary cell wall (data corresponding to Fig 1E).

RESPONSE: As we performed AFM imaging on very large portions of isolated TEs (10-20µm2), tapping imaging mode enabled to clearly distinguish between primary and secondary cell walls similarly to our observed in SEM. All the raw data have now been included in the associated excel sheet.

Point 15. Please spell out the name of all genes involved in lignin biosynthesis (CCR1, CAD4...) and briefly describe what is known about their functions.

RESPONSE: We have provided a supplemental table S2 with all the gene name, locus number, corresponding

mutant, plant species and previous reference.

Point 16. In Figs 4A and 5I, please give numbers corresponding to average circularity of each cell type x genotype to make it easier to assess differences.

RESPONSE: We agree with the reviewer and have revised Fig 4 and 5 to include convexity and statistical analyses for each cell type and genotype (circularity is as raw data in the associated excel sheet). We opted to show convexity rather than circularity as our structural equation models show that TE circularity is a consequence of its degree of inward collapse (or convexity). Clear schematic models to easily understand the relationship between circularity and convexity have been added in Fig 4B.

Reviewer #3:

Point 1. AuraLight, Seweden --> AuraLight, Sweden.

RESPONSE: changed.

Point 2. Please include the AT numbers of the A. thaliana mutants (e.g. ccoaomt1 (AT4G34050, SALK_151507; Kai et al., 2008), etc.).

RESPONSE: We have provided a supplemental table S2 with all the gene name, locus number, corresponding mutant, plant species and previous reference.

Point 3. Reference the three-point flexural tests (Nakata et al., 2020. Plant Biotechnology, 20-1214?).

RESPONSE: We thank the reviewer and have added the suggested reference in the paragraph entitled "Aliphatic and aromatic changes in lignin residues non-redundantly control distinct mechanical properties of plant stems".

Point 4. Lignin analyses: Explain the calculation (and units) of the relative values for lignin, benzaldehyde, GCHO and GCHOH. How did you measure cellulose, benzaldehydes and the P residues.

RESPONSE: Great comment, as there are no absolute quantification method for lignin (no extinction coefficient, molecular weight,… see Hatfield and Fukushima, 2005 https://doi.org/10.2135/cropsci2004.0238), we calibrated our *in situ* lignin quantification on known mutants previously shown to exhibit specific changes in lignin amount and/or composition. All cell wall biochemical parameters were estimated using *in situ* quantitative imaging using Raman spectroscopy or the Wiesner test as previously published by Blaschek et al., 2020a and 2020b.

Point 5. What are the P residues? Can your methods estimate p-hydroxyphenyl (H)-units? If not, please explain why.

RESPONSE: We can detect unsubstitued aromatic residues (call phenyl or P) using Raman spectroscopy (see Noda and Sala, 1999 and Fig S.) which derive from lignin during polymerisation (Kawamoto, 2017) and/or from side groups of non-canonical residue such as stilbenoids, flavonoids or coumaroyl esters. There are however currently no *in situ* imaging method capable of reliably and directly quantifying H residue in lignin. We have already checked and published this for Raman spectroscopy in Blaschek et al., (2020). This limitation was added in the discussion.

Point 6. Figure S1: Are the XPs and XFs (line 61) labeled as pith and fibres in Fig S1 panel C? The authors may want to use and note the abbreviations in the figure caption.

RESPONSE: We do not agree with the reviewer as the presented schematic XP and XF in Figure S1C surround TEs present in vascular bundles or secondary xylem – there are no isolated TE present in the pith.

Point 7. Looking at the size of the arrows in panel A, it seems like the sap flow is more efficient in collapsed TEs.

RESPONSE: We agree with the reviewer that our scheme was misleading and have thus revised our scheme to reduce the arrow head size in the collapsed TEs.

Point 8. Can you provide a reference to support that collapsed TEs can recover their shape?

RESPONSE: We have provided reference cited in the introduction that TEs can recover their shape (Zhang et al., 2016 - doi:10.1104/pp.16.01191).

Point 9. Figure 1: Panels A-C. Can you provide data or comment on the C/O ratios of MX TEs?

RESPONSE: We agree with the reviewer and have provided C/Cr and C/O ratio for PX and MX TEs in Fig. S2C as well as all the raw data in the associated excel sheet.

Point 10. Panels D-E. Surface adhesion seems to be increased (P=6e-04) in 50d-old MX TEs. Please revise sentence in Lines 127-129.

RESPONSE: sentence revised.

Point 11. Figure 3: Figure title: change 'herbaceous plants' for 'A. thaliana WT stems'.

RESPONSE: We agree with the reviewer and have changed "herbaceous" to "annual" plants to oppose them to "woody perennial" plants as these are two of the complementary used in our study.

Point 12. I strongly suggest to estimate the data shown in Figure 3 for WT plants also for the interfascicular fiber cells. How are the C6-C1 benzaldehyde proportions related to lignin? Are they lignin precursors/monomers?

RESPONSE: As requested by the reviewer, we have added parameters of IFs for Arabidopsis in Fig. 4. Benzaldehydes are non-canonical lignin residues (see Kim et al., 2003) and we further explained these non-canonical residues in the introduction. Note that fiber lignin composition using in situ quantitative imaging has been previously published by Blaschek et al., 2020a and 2020b.

Point 13. Lines 156-158: "Relative coniferyl alcohol (GCHOH) levels were higher in the SMX than in PX or PMX, whereas relative terminal coniferaldehyde (GCHO) residues were present in similar amounts in all three morphotypes (Fig. 3G-H)." Interestingly, this seems to suggests that the in vivo CAD activity is different in each TE morphotype. Is this data consistent with the in vitro TE morphotypes?

RESPONSE: As TE lignification depends on the cooperative supply of lignin monomers by surrounding XP (and even potential through the sap), it is difficult to relate CAD activity and TE morphotype specific lignin. However previous studies performed in Zinnia inducible cell cultures showed that CAD activity differed during TE formation (Sato et al., 1997 - 10.1104/pp.113.2.425), we have however not included this aspect in our discussion as the metabolic flow of phenylpropanoid is very complex. Note that cell wall control of GCHO for the different xylem cells has been previously published by Blaschek et al., 2020a.

Point 14. Lines 158-160: "Therefore each TE morphotype has not only specific dimensions and neighbouring cells but also specific lignin composition, amounts, structures and organisation." This sentence is not precise, the lignin monomeric composition is the same in the different TE morphotypes (as measured by S/G ratios in Panel 3E).

RESPONSE: We thank the reviewer for this comment as it highlights the widespread assumption that lignin composition can be limited to S/G ratios. We thus showed that amount of GCHO residue, ratio of GCHO/GCHOH as well as non-canonical residues significantly differ between TE morphotypes, thus showing that S/G is not sufficient to completely describe lignin composition. We elaborate on this aspect in the discussion of the present version of the manuscript to clarify the importance of lignin chemical diversity beyond S and G residues.

Point 15. Figure 4: Figure title: change 'herbaceous plants' for 'A. thaliana mutants'.

RESPONSE: We agree with the reviewer and have changed "herbaceous" to "annual" plants to oppose them to "woody perennial" plants as these are two of the complementary used in our study.

Point 16. Lines 180-183: "PX collapse was prevented by increases in lignin amount, GCHOH and terminal GCHO contents and TE perimeter (Fig. 4B). Only S residue content escalated collapse, whereas GCHO position and the proportion of neighbouring TEs had no effect (Fig. 4B)." Order the impact on the biochemical properties by their correlation values (e.g. PX collapse was prevented by increases in TE perimeter (0.47), GCHOH (0.27), lignin amount (0.2), and terminal GCHO (0.2) contents.). Please explain better what do you mean with 'only S residue content escalated collapse'. The S content doesn't seem to affect the collapse of PX, as shown in Fig 4B.

RESPONSE: We agree with the reviewer and have accordingly revised our text.

Point 17. Figures S3/S6: Please explain abbreviations and units.

RESPONSE: We agree with the reviewer and have thus added supplementary tables (S1), scheme (Fig. S3A) and references (Agarwal 2019; Blaschek et al. 2020). We also calibrated our *in situ* lignin quantification on known mutants previously shown to exhibit specific changes in lignin amount and/or composition.

Point 18. Figure S4: This figure is vaguely explained (lines 189-197).

RESPONSE: We agree with the reviewer and have accordingly revised our text "The used mutant array provided in a dataset with around 100 TEs per morphotype with a wide and continuous variation in convexity and lignin structure (Figure S5), suitable to detect specific associations between lignin amount and composition and TE resistance to collapse for each morphotype".

Point 19. Please describe: i) the impact on SMX cells, and ii) the differences between Arabidopsis and poplar. The poplar dataset has not been introduced, the authors may want to discuss this dataset later in the manuscript.

RESPONSE: We agree with the reviewer and have accordingly revised our text "Using the single cell lignin analyses of 100–170 individual TEs per morphotype and development stage, we could evaluate which parameters were associated with the capacity of specific TEs to withstand collapse using structural equation models.". We have also compared in the discussion the converging and diverging aspect of TE biomechanics and cell wall biochemistry.

Point 20. Figure caption: Interaction effects influencing vessel convexity in A. thaliana and Populus tremula x tremuloides? ...but were not omitted in the main figures for clarity--> but were omitted in the main figures for clarity

RESPONSE: sentence revised.

Point 21. Line 197: The authors should be more careful using the words "demonstrate" and "for the first time" throughout the manuscript (Lines 31, 103, 105, 143, 270, 286). Some of the conclusions are based on multiple regression analyses, I suggest them to use instead "here we show that" or "our results suggest/indicate that" and avoid 'for the first time'.

RESPONSE: sentence revised.

Point 22. Figure 5: Caption: The figure title should include poplar (e.g. Different TE morphotypes in poplar have specific morphological and chemical features associated with their resistance against collapse. Or something of the sort). Remove the typo between Populus tremula and tremuloides.

RESPONSE: caption revised to include "woody plants". We used the "x" to indicate hybrid of our poplar lines (Populus tremula×tremuloides).

Point 23. Again, here it is important to estimate the data shown for WT plants also for fiber (or ray) cells.

RESPONSE: As requested by the reviewer, we have added parameters of IFs for poplar in Fig. 5. Note that fiber lignin composition using in situ quantitative imaging has been previously published by Blaschek et al., 2020a and 2020b.

Point 24. The authors could include a supplementary figure with the chemical structures and abbreviations used for the biochemical parameters presented in the structural equation models in F4 and F5 (i.e. S, P, GCHOH, GCHO total and terminal, benzaldehydes).

RESPONSE: As requested by the reviewer, we added supplementary table S1 for the nomenclature of the lignin residues as well as Fig. S3A to show their chemical structure.

Point 25. Figure S6: Discuss similarities/differences between the CCR1-RNAi lines in poplar and the CCR-KOs in Arabidopsis.

RESPONSE: We have briefly discussed difference between *Arabidopsis* and poplar, but have refrained from focusing on single genes as we focused on the impact of changing cell wall lignin chemistry on specific TE morphotype biomechanical property for optimal sap conduction. As we cannot distinguish in our study between autocrine, paracrine and endocrine aspect of lignin formation in each TE morphotypes, we will therefore not focus on the change of specific genes but rather on a large range of chemical variation of lignin.

Point 26. Figure 6: Lines 248-250: I'm not sure if the stiffness increased ~2-fold in fah1 mutant and decreased 3-fold in the cad-double mutant. Please double check.

RESPONSE: We have checked and it is confirmed, we have moreover used different stem fragment differing in lignified cell development as previously published with apical portion of the stem mostly including TEs in contrast to basal portion which includes fibers (Hoffmann et al., 2020; Morel et al., 2022). Using this developmental gradient, we could compare the impact of turgor pressure and of lignin composition on the mechanical properties using three-point bending. We thus confirmed that the observed changes in stiffness or flexibility due to specific lignin changes mostly affected the apical portion, in line with our previous observation on TE biomechanics at the single cell level. This was added to the paragraph entitled "Aliphatic and aromatic changes in lignin residues non-redundantly control distinct mechanical properties of plant stems" and presented in Fig. 6 and S7.

Point 27. Lines 252-254: As you did in the discussion, please replace the word "controlled" by "affected", "modulated" or "changed".

RESPONSE: We have accordingly changed some of these wording when we judge appropriate.

Point 28. Remove "significantly" Lines (240, 248, 249, 251,..)

RESPONSE: We have removed some although they refereed to statistical relevant data.

Point 29. Figure 7: Lines 268-270: "Overall our results showed that the increased flexibility conferred by GCHO residues to TEs enables plants to better resist the extreme Ψ changes caused by drought. We therefore demonstrated that lignin structure directly controls the hydraulic properties of TEs to cope with environmental changes" This is an overstatement, please rephrase. Some references suggest that this may be related to enhanced stomatal closure (https://doi.org/10.1093/treephys/tpx093).

RESPONSE: We have accordingly revised the sentence and added reference for stomatal closure (Martin-StPaul et al., 2017 - doi:10.1111/ele.12851).

Point 30. Discussion: "Completely revises the 'random' lignification of plant cells to a genetically controlled process". The authors must be more careful here too. Although some enzymes have been proposed to initiate lignin formation, it is very likely that at least part of the polymerization process involves free oxidative radical coupling of monolignols.

RESPONSE: We agree with the reviewer and have accordingly removed any mention of this as we did not provide any information on phenoloxidases.

Point 31. The authors could speculate here on the potential biological roles of the lignin residues in fiber cells.

RESPONSE: We prefer not to speculate on the role of lignin chemistry in fibers as our data focused on tracheary elements. We nevertheless provided additional data using different stem segments (each showing differences in fiber lignification) that confirmed that the main biomechanical differences between genotypes depended mostly on TEs.

Point 32. Abstract: The abstract has to explain in more detail what are the non-redundant biological roles of the different lignin residues.

RESPONSE: We have accordingly completely revised the abstract.

TPC2021-RA-00813 1st Editorial decision – *revision requested* June 5, 2022

We have received reviews of your manuscript entitled "Plant biomechanics and resilience to environmental changes are controlled by specific lignin chemistries in each vascular cell type and morphotype." Thank you for submitting your best work to The Plant Cell. The editorial board agrees that the work you describe is substantive, falls within the scope of the journal, and may become acceptable for publication, pending revision and potential re-review.

We ask you to pay attention to the following points in preparing your revision:

As you will see from the reviews below, Reviewers 2 and 3 are still not convinced. It will be important to do additional experiments and/or clarify your results, limit your conclusions, and discuss other confounding factors and interpretations of the data.

--- Reviewer comments:

TPC2021-RA-00813R1 1st Revision received July 23, 2022

Reviewer comments on previous submission and **author responses**:

Reviewer #1

This new version of the manuscript has been greatly improved. The authors addressed all the point I previously raised. I recommend this article for publication.

Point 1. In lines 143-146: "Complementary biochemical analyses confirmed that the post-mortem changes in cell wall corresponded to increases in lignin concentrations rising gradually only in TE cells to ~25% of total dry cell wall weight by day 30 (Fig. S2)." I cannot see where the % of total dry cell wall weight is indicated in Fig. S2.

RESPONSE: We thank the reviewer for noticing this issue as the units (mg of TGA lignin/g of cell walls) were missing, thus leading by day 30 up to 25% of lignin in the cell walls of TEs, which is now revised in Figure S2.

Point 2. In lines 189-192: "The ratio of terminal coniferaldehyde (GCHO) to total coniferyl alcohol (GCHOH ; including terminal coniferyl alcohol as well as internal guaiacylglycerol, pinoresinol and other incorporated GCHOH structures) was higher in the SX than in PX or MX (Fig. 3F)." The ratio is lower in the SX than in PX or MX according to Fig. 3F.

RESPONSE: We thank the reviewer for noticing this typo and have accordingly revised it to define that SX have significantly lower terminal coniferaldehyde to total coniferyl alcohol ratio that PX or MX. We thus accordingly modified (and shown using track-changes) the paragraph entitled "Each TE morphotype differs in morphology, lignin concentration and composition in annual plants."

Point 3. The Figure S9C could be incorporated in the main Fig. 7 as it is a major result.

RESPONSE: We thank the reviewer for this suggestion and have accordingly added to Figure 7 the panel C of Figure S9.

Reviewer #2:

The authors have addressed most of my concerns. I still have have one major point. The results from 3-point bending test question the link between lignification and mechanical properties:

Point 1. The results for WT are not consistent between Figs 6C and 6F

RESPONSE: We thank the reviewer for this comment and added in the text and figure legends the reasons behind the slight differences of ranges in WT stem segments properties in air which were due to a slight different stem ages and growth instance between our two three point-bending experiments. Note that between the two experiments, air-treatment of each different stem segment of WT plants still presented overlapping stem diameters, flexural strength, stiffness and flexibility – they only differed in the range of the data spread due to different number of replicates (from 6 to 8 for incubation experiment and from 14 to 18 for the genotype comparison). This slight

difference in range nevertheless did not affect our conclusion between genotypes. We thus accordingly modified (and shown using track-changes) the paragraph entitled "Aliphatic and aromatic changes in lignin residues non-redundantly control distinct mechanical properties of plant stems" as well as in Figure 6 legend.

Point 2. The statement lines 299-300 that mutants 'affected more in apical than middle and basal stem segments' is not supported by the data.

RESPONSE: We thank the reviewer for noticing this slight overstatement as indeed only the flexibility but not the stiffness affected more the apical than middle and basal stem segments, we have thus revised the sentence to specific that only the coniferaldehyde over-accumulating plant exhibited greater flexibility in its apical portion. We thus accordingly modified (and shown using track-changes) the paragraph entitled "Aliphatic and aromatic changes in lignin residues non-redundantly control distinct mechanical properties of plant stems".

Point 3. All mutants are distinct from WT whatever the developmental stage. This rather supports the conclusion that differences with WT are not related to lignification.

RESPONSE: We thank the reviewer for this comment. As no difference were observed in stem growth rate nor in stem xylem organization by ourselves but also by others using kinetic analyses (for fah1 - Vanholme et al., 2012 - 10.1105/tpc.112.102574 / for cad4xcad5 - Thevenin et al., 2011 - 10.1093/mp/ssq045), the main difference between the genotypes tested is therefore their lignin composition. We have thus shown that independently of the developmental stage, (i) it is not the water content but the cell walls that altered the stem biomechanical properties, and (ii) for similar water content, genotypes with altered lignin chemistry had significantly different stem biomechanics. We moreover made all our flexural measurements to be independent from stem diameter (and thus development) using the Euler-Bernoulli's equation (please see materials and methods section). We nevertheless accordingly modified (and shown using track-changes) the paragraph entitled "Aliphatic and aromatic changes in lignin residues non-redundantly control distinct mechanical properties of plant stems" to highlight the above-mentioned points.

Reviewer #3:

The manuscript by Menard et al. details an interesting integrated biomechanical, genetic and molecular modeling approach to interrogate the biological function of lignin in different cell types. This is a relevant research question with broad biotechnological applications in plant biology. The techniques used here are novel, and the quality of the experiments is excellent. Their analyses show convincing evidence that lignin content and composition impact the mechanical properties of different cell types by using both Arabidopsis cell suspension cultures and whole plants in Arabidopsis and poplar. However, this reviewer considers that the study still requires additional experiments to prove the suggested role of coniferaldehyde on the increased flexibility and drought tolerance of the Arabidopsis cad4/cad5 double knockout mutants.

Point 1. If coniferaldehyde enable plants to tolerate drought, the WT plants watered with certain concentration of coniferaldehyde may perform better under drought stress. These plants could also have increased flexibility and reduced stiffness in the three-point flexamural tests. Plants watered with coniferyl alcohol can be used as controls.

(Reviewer 3 clarified in the consultation: "I believe that considering the lignin coniferaldehyde residues in the vascular bundles as the major factor controlling stem flexibility and drought tolerance in the cad double mutants is not fully supported by the data. To test this hypothesis, I suggest rewatering or spraying water-stressed WT plants with different concentrations of exogenous coniferaldehyde. If the results are negative, the authors may consider the total lignin content, S:G ratios, or other cell wall properties (of TEs or interfascicular fibers) as potential additional factors contributing stem flexibility and drought stress tolerance in lignin-modified plants.")

RESPONSE: We thank the reviewer for this comment and suggestion to improve our experiments to define the relationship between drought tolerance and lignin composition. The proposed experiment is indeed an exciting idea and would – if feasible – provide very useful insight. However, the reviewer's suggestion to externally supply monomeric solutions of cell wall polymers (or even their glucoside form) by directly watering or spraying plants to change their cell wall composition is not possible on intact fully grown plants. Moreover, external supply of lignin monomers will affect plants as (i) large overaccumulation of coniferyl alcohol or other phenylpropanoids have toxic effects on plants (Väisänen et al., 2015 - 10.1007/s00425-015-2348-7; Perkins et al., 2020 - 10.1002/pld3.265), (ii) coniferyl alcohol or other phenylpropanoids have bioactive properties altering plant development (Väisänen et al., 2015 - 10.1007/s00425-015-2348-7; El Houari et al., 2021 - 10.3389/fpls.2021.734070), (iii) coniferaldehyde and coniferyl alcohol can be interconverted by CADs (please see Tsuji et al., 2005 - 10.1007/S00425-005-1517-5 – who show that feeding coniferyl alcohol glucosides to cut stems results in an increase of coniferladehyde), and (iv) coniferaldehyde can inhibit CAD specialized in the conversion of cinammyl alcohols as well as change the phenylpropanoid metabolism (Wang et al., 2014 - 10.1105/tpc.113.120881), without considering that (v) free diffusible access to xylem tissues will be hindered by epidermal cuticle (enriched in phenolics) in stems and Casparian strip (composed of lignin) in roots. Lastly, even if external supply of coniferyl alcohol or coniferaldehyde did not have the above listed effects and could be directly incorporated into lignin, we would not be able to control if the added monomers incorporate within the lignin polymers or at its termini. The position of the residue in the

polymer has an important effect as we have shown using our imaging analysis and our molecular dynamic simulation (Tab. 1 and Fig. S11). We nevertheless did not mention any of these above arguments that do not support the directed changes of lignin composition using external feeding as we did not attempt such experiments.

Our study did not limit stem flexibility and drought recovery to only the proportion of aldehyde residues in lignin. It includes multiple complementary experiments showing that lignin amount, S/G, alcohol/aldehyde, internal to terminal aldehyde and the presence of non-canonical residues all contribute to different extent (depending on the morphotypes and their post-mortem maturation stage) to the biomechanics of TEs not to inwardly collapse under normal and/or extreme negative pressure. As our analyses showed that lignin amount and S/G were important, we focused on alcohol to aldehyde that is a novel aspect of lignin not previously studied. It is important to consider that all vascular plants, even the ones devoid of S residues like the conifers (which only have TEs in their xylem), have the capacity to resist drought that also induces TE collapse (Cochard et al., 2004 - 10.1104/pp.103.028357). From the evolutionary point-of-view, we believe it is more likely that the control of lignin biomechanical properties in sap-conducting TEs was fine-tuned first using alcohol/aldehyde and later using S/G. We thus revised the text accordingly in the discussion section.

Nevertheless, we understand the need to support our drought recovery experiments with additional data to clearly compare lignin alcohol/aldehyde to S/G. We therefore added (i) new drought results to extend our WT to cad4xcad5 comparison with fah1 as well as cad4xcad5xfah1 (previously biochemically characterized by Anderson et al., 2015 - 10.1104/pp.15.00294), and (ii) molecular dynamic simulation of β-O-4 interlinked sinapyl alcohol heptamer (with an α-OH) using the same set-up as used for our other heptamers. These new results showed that (a) the removal of S residues in fah1 did not change the drought recovery compared to WT, (b) the removal of S residues in cad4xcad5xfah1 did not change the drought recovery compared to cad4xcad5 that remained greater than WT and fah1, and (c) the heptamers of sinapyl alcohol showed compaction and Young modulus similar to coniferyl alcohol (thus much greater than coniferaldehyde). Altogether, these new results show that TE recovery from drought is more dependent on aldehyde/alcohol rather than on S/G, this result is in-line with TE lignin composition (enriched in G residues) as well as evolutionary constraints. Results obtained were added to Fig. 7 for the drought recovery and Tab. 1 and Fig. S11 for the dynamic molecular simulations as well as described in the paragraphs entitled "Increasing coniferaldehyde residues in lignin changes molecular conformations, torsions and reduces stiffness compared to coniferyl alcohol residues" and "Increased TE cell wall flexibility due to coniferaldehyde in lignin enables plants to better recover from drought".

TPC2021-RA-00813R1 2nd Editorial decision – *acceptance pending* August 24, 2022

We are pleased to inform you that your paper entitled "Plant biomechanics and resilience to environmental changes are controlled by specific lignin chemistries in each vascular cell type and morphotype" has been accepted for publication in The Plant Cell, pending a final editorial review by a science editor. At this stage, your manuscript will be evaluated by a science editor with respect to its presentation of scientific content, compliance with journal policies, and presentation for a broad readership.

Final acceptance from Science Editor Sept. 11, 2022
